# Polydatin combined with hawthorn flavonoids alleviate high fat diet induced atherosclerosis by remodeling the gut microbiota and glycolipid metabolism

**DOI:** 10.3389/fphar.2025.1515485

**Published:** 2025-03-03

**Authors:** Dan Li, Yujuan Li, Shengjie Yang, Xiaonan Zhang, Yu Cao, Ran Zhao, Yixi Zhao, Xiao Jin, Jing Lu, Xinyue Wang, Qiutao Wang, Longtao Liu, Min Wu

**Affiliations:** ^1^ Guang’an Men Hospital, China Academy of Chinese Medical Sciences, Beijing, China; ^2^ The Dongfang Hospital of Beijing University of Chinese Medicine, Beijing, China; ^3^ Xiyuan Hospital, China Academy of Chinese Medical Sciences, Beijing, China; ^4^ Aerospace Center Hospital, Beijing, China; ^5^ Graduate School, Beijing University of Chinese Medicine, Beijing, China

**Keywords:** atherosclerosis, gut microbiota, TMAO, polydatin, hawthorn flavonoids

## Abstract

**Background:**

Atherosclerosis is a widely studied pathophysiological foundation of cardiovascular diseases. Inflammation and dyslipidemia are risk factors that promote the formation of atherosclerotic plaques. The gut microbiota and their metabolites are considered independent risk factors for atherosclerosis. Polydatin combined with hawthorn flavonoids, as the extracts of *Polygonum cuspidatum Sieb. et Zucc*. and *Crataegus pinnatifida Bunge*, have shown excellent cardiovascular protective effects. However, the underlying mechanism requires further investigation. Our study aimed to explore the anti-atherosclerotic mechanism through gut microbiota and their metabolites.

**Methods:**

ApoE^−/−^ mice were fed either a normal-chow diet or a high-fat diet. The polydatin combined with hawthorn flavonoids group received varied doses of polydatin and hawthorn flavonoids: a high dose (polydatin 200 mg/kg daily; hawthorn flavonoids 100 mg/kg daily), a medium dose (polydatin 100 mg/kg daily; hawthorn flavonoids 50 mg/kg daily), and a low dose (polydatin 50 mg/kg daily; hawthorn flavonoids 25 mg/kg daily). The control and model groups were administered distilled water (0.2 mL daily). The experiment lasted for 24 weeks.

**Results:**

Polydatin combined with hawthorn flavonoids administration significantly reduced lipid and inflammatory cytokine levels, meanwhile, the atherosclerotic lesions in a high-fat diet-induced ApoE^−/−^ mice were significantly decreased. Additionally, polydatin combined with hawthorn flavonoids also inhibited the enhancement of trimethylamine N-oxide (TMAO), trimethylamine (TMA) levels of HFD-induced ApoE^−/−^ mice by regulating the expression of hepatic flavin-containing enzyme monooxygenase 3 (FMO3). 16S rRNA sequencing results demonstrated that high-dose polydatin combined with hawthorn flavonoids treatment increased the abundance of *Actinobacteriota*, *Atopobiaceae* and *Coriobacteriaea_UCG-002*, and decreased the abundance of *Desulfobacterota*. *Norank_f_Muribaculaceae* was enriched in the medium-dose polydatin combined with hawthorn flavonoids and simvastatin groups, and *Lactobacillus* was mainly increased in the simvastatin and the low-dose polydatin combined with hawthorn flavonoids groups. According to the metagenetic results, functional annotations also suggested that the biological processes of each group mainly focused on metabolism-related processes. Specifically, polydatin combined with hawthorn flavonoids may regulate the abundance of TMA-producing bacteria (Coriobacteriaceae, *Desulfovibrio*, *Muribaculum*, and *Clostridium*) and related enzymes in glycolipid metabolic pathways to exert an important effect on the prevention of atherosclerosis.

**Conclusion:**

Our results suggested that polydatin combined with hawthorn flavonoids could regulate the glucolipid metabolism-related pathway, attenuate inflammatory cytokine levels, and reduce atherosclerotic plaques by remodeling gut microbiota.

## 1 Introduction

Cardiovascular diseases (CVDs) are the leading cause of mortality worldwide (Bansilal et al., 2015). Atherosclerosis is one of the pathological basis of CVDs and is characterized by chronic inflammatory artery disease accompanied by disturbances in glucose and lipid metabolism (Joris and Gloor, 2019). Inflammation spreads throughout the process of atherosclerosis, and in combination with lipid deposition, aggravates the progression of atherosclerotic plaques (Blagov et al., 2023). Risk factors, including dyslipidemia and diabetes, promote atherosclerosis progression (Vaccarezza and Galassi, 2023). There has been increasing evidence that gut microbiota can regulate gut microbial homeostasis in the human body (Zhou et al., 2020). The gut microbiota is responsible for converting different complex substances into bioactive metabolites (Lau et al., 2017) and is considered an indicator of host pathology, including CVDs (Wang and Zhao, 2018). Thus, on one hand, gut microbiota may indirectly affect atherosclerosis by increasing the risk of glucose and lipid metabolism disorders, such as obesity and diabetes (Traughber et al., 2023); on the other hand, gut microbiota influences atherosclerosis by enhancing the direct risk factors of atherosclerotic lesions through its metabolites (Ma and Li, 2018).

Trimethylamine-N-oxide (TMAO) is generated from dietary sources, which are widely present in high-protein diets and dairy products (Koeth et al., 2019). Intriguingly, trimethylamine (TMA)-producing bacteria, a type of specific gut bacteria (Rath et al., 2019), may be involved in the metabolism of these foods by producing TMA-lyse [mainly choline-TMA lyase (CutC/D)] (Simó and García-Cañas, 2020), and the oxidation of the hepatic flavin-containing enzyme monooxygenase 3 (FMO3) also participates in the microbial pathways involved in TMA/TMAO formation (Schugar and Brown, 2015). Recent studies have reported that choline utilization (cut) gene clusters, such as *Lachnoclostridium*, *Desulfovibrio*, and *Clostridium*, promote the conversion of choline to TMA, which is associated with elevated TMAO levels (Cai et al., 2022). The gut microbial alterations by TMA-producing bacteria are often accompanied by elevated TMAO levels, plaque lipid deposition, high lipid levels, and damaged cholesterol transport (Luo et al., 2022). In addition, inflammatory cytokines have been highlighted as a central driving force that is triggered by TMAO (Singh et al., 2017) and aggravate genes related to gluconeogenesis and glucose transport (Gao et al., 2015), thus indicating the microbial mechanism of atherosclerosis (Saaoud et al., 2023). Collectively, the gut microbiota, acting as a virtual endocrine organ (Qi et al., 2021), accompanied by TMAO, contribute to the pathologic processes of atherosclerosis through various mechanisms, mainly via inflammation (Bolte et al., 2021) and glycolipid metabolism disorders (Massey and Brown, 2021). These effects occur when there is an imbalance occurs in the host’s gut microbiota.

It is widely known that statins may prevent the progression of atherosclerosis to a certain extent. However, myopathy, renal disease, rhabdomyolysis, and other muscle-related adverse diseases associated with statins should not be overlooked. It has been shown that natural compounds may confer protection against atherosclerotic cardiovascular disease (ASCVD) through various mechanisms (Arauna et al., 2019). Especially, many plant compound are beneficial for metabolic diseases, such as CVD, obesity, and diabetes, which are caused by the metabolic disorder of fat, sugar, and protein (Wang et al., 2022). emerging evidence has indicated that paeonol prevents the development of atherosclerosis by suppressing the release of inflammatory cytokines (Li et al., 2009).

Modern pharmacological studies (Qu et al., 2021) and clinical trials (Zifu et al., 2022) have confirmed that specific Chinese herbs or extracts have shown excellent potential therapeutic effects in ASCVD owing to their prominent detoxifying and blood-activating effects (Xue et al., 2013). *Polygonum cuspidatum Sieb. et Zucc*. (*P. cuspidatum*) and *Crataegus pinnatifida Bunge* (*C. pinnatifida*) as representative detoxifying and blood-activating herbs (Qin et al., 2022), are known for their efficacy in blood activation and resolving stasis, clearing heat, and detoxification (Zhu et al., 2018a). “Stasis” and “poison” in traditional Chinese medicine are not only pathogenic factors, but also pathological products of zang-fu dysfunction. “Stasis poison” consists of two elements including “stasis” and “poison,” which interact and influence each other. Toxic heat obstructed in the blood vessels is easy to become blood stasis. The application of activating blood and detoxification in AS has become increasingly extensive. In the Qing Dynasty, Wang Qingren summarized the theory of stasis and established detoxification and blood-activating decoction. *P. cuspidatum* as one of the representative Chinese herbs for promoting blood circulation, clearing heat, removing dampness and relieving pain, was first recorded in “Mingyi Bielu”. *P. cuspidatum* is often used in TCM to clear dampness and heat, and detoxifying, promoting blood circulation, and removing blood stasis (Wu et al., 2020). It was mentioned first for“treating dysentery” in *Tang Materia Medica* (*Tang Ben Cao*) dating back to 659 AD, the first known official pharmacopeia in the world (Liu et al., 2011). Moreover, it acts on tonifying the spleen to promote digestion and activate blood circulation to dissipate blood stasis.

Extensive evidence has shown that polydatin (Zhao et al., 2022) or hawthorn flavonoids (Hu et al., 2022) could exert cardioprotective effects and alleviate metabolic disorders, partially via the gut microbiota. Previously, our team combined hawthorn to promote blood circulation and resolving blood stasis with *P. cuspidatum* to clear heat and detoxify (Feng et al., 2011), applied it to the treatment of acute coronary syndrome, and achieved great clinical efficacy (Wu et al., 2021). In addition, our previous clinical trial also confirmed the remarkable anti-atherosclerotic efficacy of polydatin (the extract of *P. cuspidatum*) combined with hawthorn flavonoids (the extract of hawthorn) (Liu et al., 2014). However, the intimate associations and mechanisms were unknown. Therefore, we hypothesized that the anti-atherosclerotic mechanism of polydatin combined with hawthorn flavonoids (PH), compatibility of traditional Chinese medicines, is linked with the inhibition of TMAO (Li and Tang, 2017), related pathways, and the regulation of gut microbiota disorders (Trøseid et al., 2020). Taking these factors into consideration, we evaluated the indicators of lipids, inflammation, and plaque area, provided an integrated metagenomic sequencing (Zhulin, 2016), and targeted metabolomics (Fiehn, 2016) analysis of the metabolite TMAO to further explore the specific therapeutic mechanism of PH.

## 2 Materials and methods

### 2.1 Drugs and diets

Polydatin (purity ≥98%, 140 g, PO210425, Xi’an Guanjie BioTech Co. Ltd., Xi’an, China) and hawthorn flavonoids (purity ≥90%, no. AKH15-2; Linyi Aikang Pharmaceutical Co., Ltd., Shandong, China) were mixed to prepare a solution. simvastatin was purchased from Hangzhou MSD Pharmaceutical Co. Ltd. (20 mg, U010049; Zhangzhou, China). All the natural products using from a single manufacturing batch. The drug dosage standard for this experiment was in accordance with the First edition of Chinese Pharmacopoeia 2015. The clinical recommended daily oral doses for adults were 15 g knotcane and 12 g hawthorn. The dosage for mice was converted according to the equivalent dose ratio of adult (adult weight calculated by 60 kg) to the body surface area of mice and the content percentage of Chinese medicine components in Chinese medicinal materials. The high-fat diet (HFD) containing 21% saturated fat, 0.15% cholesterol and 1% choline was purchased from Keao Xieli Feed Co., Ltd. [SCXK (Beijing) 2019–0003].

### 2.2 Animal experiments

Specific pathogen-free (SPF) male C57BL/6J mice (8-week-old, 19–21 g) and male apolipoprotein E knockout (ApoE^−/−^) mice of the same age and genetic background were obtained from Vital River Laboratory Animals (Beijing, China). The mice were bred in a SPF laboratory and had free access to food and water. After 13 weeks, aortic atherosclerotic plaque formation was confirmed by microscopic observation after dissection, indicating that the atherosclerotic model was successfully prepared. Subsequently, all mice were randomly assigned to six groups (n = 10 in each group): (Bansilal et al., 2015): control: C57BL/6J mice received normal diet; (Joris and Gloor, 2019); model: ApoE^−/−^ mice received HFD; (Blagov et al., 2023); low dose of PH group (PHL): HFD supplemented with polydatin (50 mg/kg daily) and hawthorn flavonoids (25 mg/kg daily); (Vaccarezza and Galassi, 2023); medium dose of PH group (PHM): HFD supplemented with polydatin (100 mg/kg daily) and hawthorn flavonoids (50 mg/kg daily); (Zhou et al., 2020); high dose of PH group (PHH): HFD supplemented with polydatin (200 mg/kg daily) and hawthorn flavonoids (100 mg/kg daily); (Lau et al., 2017); simvastatin: HFD supplemented with simvastatin (5 mg/kg daily). The control and model groups received distilled water (0.2 mL daily). The experiment lasted for 24 weeks, and all mice were fasted overnight and euthanized using isoflurane inhalation at the end of the experiment. The experimental protocol was approved by Institutional Animal Care and Use Committee (IACUC), Guang’an men Hospital, China Academy of Chinese Medical Sciences. The protocol for *in vivo* studies was approved by the Ethics Committee of Guang’anmen Hospital, China Academy of Chinese Medical Sciences (IACUC-GAMH-2021-013).

### 2.3 Histology

After collection of blood samples, the circulatory system was rinsed with normal saline. The thoracic aorta was isolated, and adherent fat was removed. The heart and aorta were perfused with phosphate-buffered saline for 10 min and then with 3.5% paraformaldehyde for 5 min. The heart was then rapidly removed, fixed in 10% buffered neutral formalin for 24 h, and washed in tap water to remove the formalin. For enface staining of the plaque areas in the mice, the whole aorta was soaked with Oil Red O (ORO) solution and photographed against a black background. The aortic sinus specimens were fixed with 4% paraformaldehyde, dehydrated in layers, and embedded in paraffin wax. Continuous sections (5 μm) were taken from the aortic sinus to the aortic arch at intervals of 40 mm, and atherosclerotic aortic sinus lesions were evaluated using hematoxylin-eosin (H&E) staining. The remaining aortic sinus samples were buried at the optimal cutting temperature, and frozen sections were used for ORO staining to evaluate lipid deposition areas. The ratio used to make a working dilution of the red O dye was 3:2 (red O dye:distilled water). The ratio of isopropanol to distilled water was 3:2, which formed a 60% isopropanol solution. Subsequently, the aorta was removed from the 10% formalin solution, rinsed for 1–2 min with distilled water, dipped in 60% isopropanol for 1 min, stained with the oil red O working dilution for 10 min, and then the aorta was placed in 60% isopropanol. Aortas were differentiated until the vascular wall of the aortas was transparent, and the difference between the color and the plaque was clear. Finally, the aortas were stored in 10% formalin, after staining and taking photos. Three lesion areas were compared by using computer-supported morphometry (ImageJ software) at 30-μm intervals, and the average lesion size was estimated. The percentage of plaque in total vascular area represented the relative severity of AS.

### 2.4 Lipid measurements

The plasma concentrations of cholesterol (TC), triglycerides (TG), low-density lipoprotein cholesterol (LDL-C), very low-density lipoprotein cholesterol (VLDL-C), and high-density lipoprotein cholesterol (HDL-C) were measured using an automatic bioinformatic instrument (BackmanAU5821, United States).

### 2.5 AimPlex multiplex immunoassays of inflammatory cytokines

Serum levels of interleukin-1-beta (IL-1β), interleukin-2 (IL-2), interleukin-6(IL-6), interleukin-17A (IL-17A), tumor necrosis factor alpha (TNF-α) and hs-CRP were measured using the Aimplex Mice Custom Premixed Analyte kit (Beijing Quantobio, China).

### 2.6 Immunological biomarkers analysis

Paraffin-embedded fixed aortic arch tissues were sliced into approximately 10 μm sections. These sections were incubated with nuclear factor-кB (NF-κB) p65 Rabbit PolyClonal antibody (Proteintech; 10745-1-AP, 1:500); tumor necrosis factor alpha (TNF-α) antibody (Proteintech; 60291-1-Ig, 1:1,000); and Anti-FMO3 Antibody (abcam; ab126711, 1:5000) for overnight at 4°C. After being washed, slices were incubated with horseradish peroxidase (HRP)-conjugated anti-rabbit secondary antibody (Proteintech; SA00001-2, 1:300) for 1 h at 37°C. Hematoxylin was added to the slices for 30 s and the slices were counterstained after washing with PBS. The area of positive staining represented the levels of NF-κB and TNF-α in the aortic arch tissue, which was calculated and evaluated using the ImageJ software.

### 2.7 Western blot

Protein concentrations in the aorta and liver tissues were quantified using a BCA kit (Thermo Scientific). Proteins were separated by 5% or 10% sodium dodecyl sulfate-polyacrylamide gel electrophoresis and transferred onto polyvinylidene fluoride membranes (Millipore, Burlington, MA). The membrane was blocked with 5% skimmed milk, for 1 h, and then incubated with primary antibody overnight at 4°C: TNF-α, NF-κB p65 and Anti-FMO3 antibody were same as the above (as shown in 2.6); β-actin (ABclonal, AC026, 1:1,000). The blots were incubated with HRP-conjugated anti-rabbit IgG for 1 h. After washing, the membranes were analyzed using chemiluminescence detection kit.

### 2.8 Quantitative RT-PCR

Total RNA was extracted from liver tissues using TRIzol reagent. The cDNA was synthesized using SuperScript II (TaKaRa Bio). RT-PCR for mRNA amplification was performed using a SYBR Green Master Mix kit. Details of the real-time PCR conditions are shown in Supplementary Table S1. The target mRNA level was normalized to the β-actin level. All primer sequences were designed using the PrimerBank software and are listed in Supplementary Table S2.

### 2.9 TMA and TMAO detected by HPLC-MS/MS

Serum TMAO and TMA levels were measured using the Waters ACQUITY ultra-high-performance liquid chromatography system (Waters Corporation, Milford, MA, United States). Samples were separated using a mobile phase gradient consisting of a mixture of 5 mM ammonium formate as solvent A and acetonitrile as solvent B. The analytes were eluted using the following gradient profile: 90% B, 10% A for 1 min; 3.5 min, 50% A, 50% B; and reconditioning at 90% B, 10% A for 8.6 min. The detailed contents of the mobile phase gradient and mass spectrometry conditions are shown in Supplementary Tables S3, S4. Data processing was completed using the TargetLynx software.

### 2.10 DNA extraction and 16S rRNA sequencing

Bacterial DNA was extracted from 0.5 g fecal samples utilizing the FastDNA^®^ Spin Kit for SoilDNA (Qiagen, Limburg). DNA integrity was detected using 1% agarose gel electrophoresis, and the concentration and purity of DNA were detected using a NanoDrop 2000 microspectrophotometer (Thermo Fisher Scientific, United States). The forward primer 338F (5′-ACT​CCT​ACG​GGA​GGC​AGC​AG-3′) and reverse primer 806R (5′-GGACTACHVGGGTWTCTAAT-3′) were used to amplify the V3-V4 variable region of the bacterial 16S rRNA gene. Amplification was performed according to the standard protocols. PCR products were detected by 2% agarose gel electrophoresis and purified using an AxyPrep DNA Gel Extraction Kit (Invitrogen, Carlsbad, CA, United States). A NEXTFLEX Rapid DNA-Seq Kit (Bioo Scientific, United States) was used to prepare the sequencing library. The amplified products were collected and sequenced on an Illumina MiSeq PE300/NovaSeq PE250 platform (Illumina, San Diego, United States). All raw sequences have been submitted to the Sequences Read Archive database at the NCBI under accession number PRJNA1122934.

### 2.11 Biochemical analyses

The quality control and splicing process of the raw sequencing sequences were based on the fastp software that was merged with FLASH. The UPARSE software was used to cluster operational taxonomic units (OTUs) with a 97% similarity cutoff. Each sequence was classified using the RDP classifier and compared to the Silva 16S rRNA database (V128) at a threshold of 70%. Alpha diversity and beta diversity analysis was used to show the distribution and similarity using principal coordinate analysis (PCoA). The differences at different taxonomic levels were evaluated using linear discriminant analysis effect size (LEfSe). A correlation heatmap was utilized to visualize the relationships between the different species and other parameters.

### 2.12 Metagenome sequence and taxonomic annotation

Quality control and optimization were performed using software fastp, and filtered reads were assembled (contigs ≥300 bp). In addition, open reading frames (ORFs) prediction and translation of contigs were performed using MetaGene to obtain the corresponding amino acid sequences. CD-HIT was used to cluster the predicted genetic sequences, and the non-redundant gene set was constructed. Diamond was used to align the amino acid sequences with the GENES database using BLASTP (version 2.2.28) and annotate the kyoto encyclopedia of genes and genomes (KEGG) databases (version 94.2). The amino acid sequences were aligned to the carbohydrate-active enzymes (CAZymes) database using HMMER (version 3.1b2). The abundance and differences in the corresponding species, CAZy, and KEGG functional categories were estimated using normalized RPKM values.

### 2.13 Statistical analyses

Statistical analyses were performed using GraphPad Prism 9.0. All data were presented as the mean ± standard deviation (SD), and multiple comparisons were analyzed using ANOVA. Statistical significance was set at *P* < 0.05.

## 3 Results

### 3.1 Polydatin and hawthorn flavonoids (PH) reduced body weight and lipid levels in HFD-fed ApoE^−/−^ mice

**FIGURE 1:**
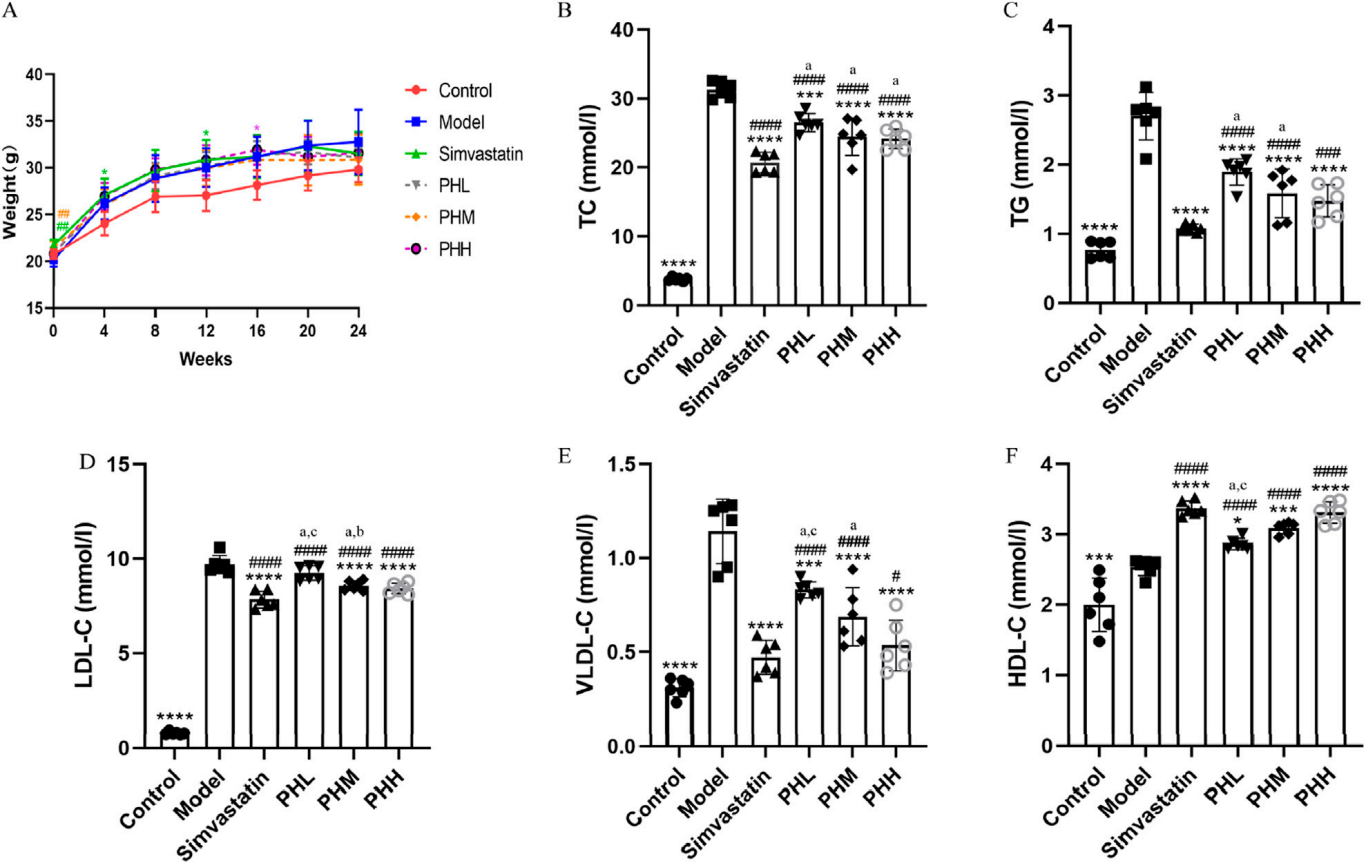
PH alleviated serum lipid levels in ApoE^−/−^ mice. **(A)** Body weight. **(B)** TC. **(C)** TG. **(D)** LDL-C. **(E)** VLDL-C. **(F)** HDL-C. All data are shown as the mean ± SD (n = 6). *P < 0.05, **P < 0.01, ***P < 0.001, ****P < 0.0001, vs. Model; ^#^ *P* < 0.05, ^##^ *P* < 0.01, ^###^ *P* < 0.001, ^####^ *P* < 0.0001, vs. Control; ^a^ *P* < 0.05 vs. simvastatin; ^b^ *P* < 0.05 vs. PHL; ^C^ *P* < 0.05 vs. PHH.

Throughout the experiment, an obvious increase in body weight was observed in HFD-fed mice. After 11-week of intervention, the PH and simvastatin groups may have experienced a lower gain in body weight than that of the model group (Figure 1A). In addition to the possible anorexic effect that may be induced by HFD, supplementation with PH or simvastatin may also have exerted certain effects on the body weight and metabolic function of mice. Compared to the control group, a remarkably increasing trend of TC, TG, LDL-C, and VLDL-C was observed in HFD-fed mice (*P* < 0.001). Whereas mice supplemented with PH or simvastatin exhibited low levels of TC, TG, LDL-C, and VLDL-C, accompanied by high levels of HDL-C, reflecting the hyperlipidemia caused by the HFD; these levels were reversed by PH or simvastatin administration (Figures 1B–F). These results, together with the increase in body weight, highlight the synergistic effect of lipid metabolism as a potential modulator of atherosclerosis and the important role of PH on the treatment of dyslipidemia.

### 3.2 PH attenuates HFD-induced atherosclerosis in HFD-fed ApoE^−/−^ mice

**FIGURE 2:**
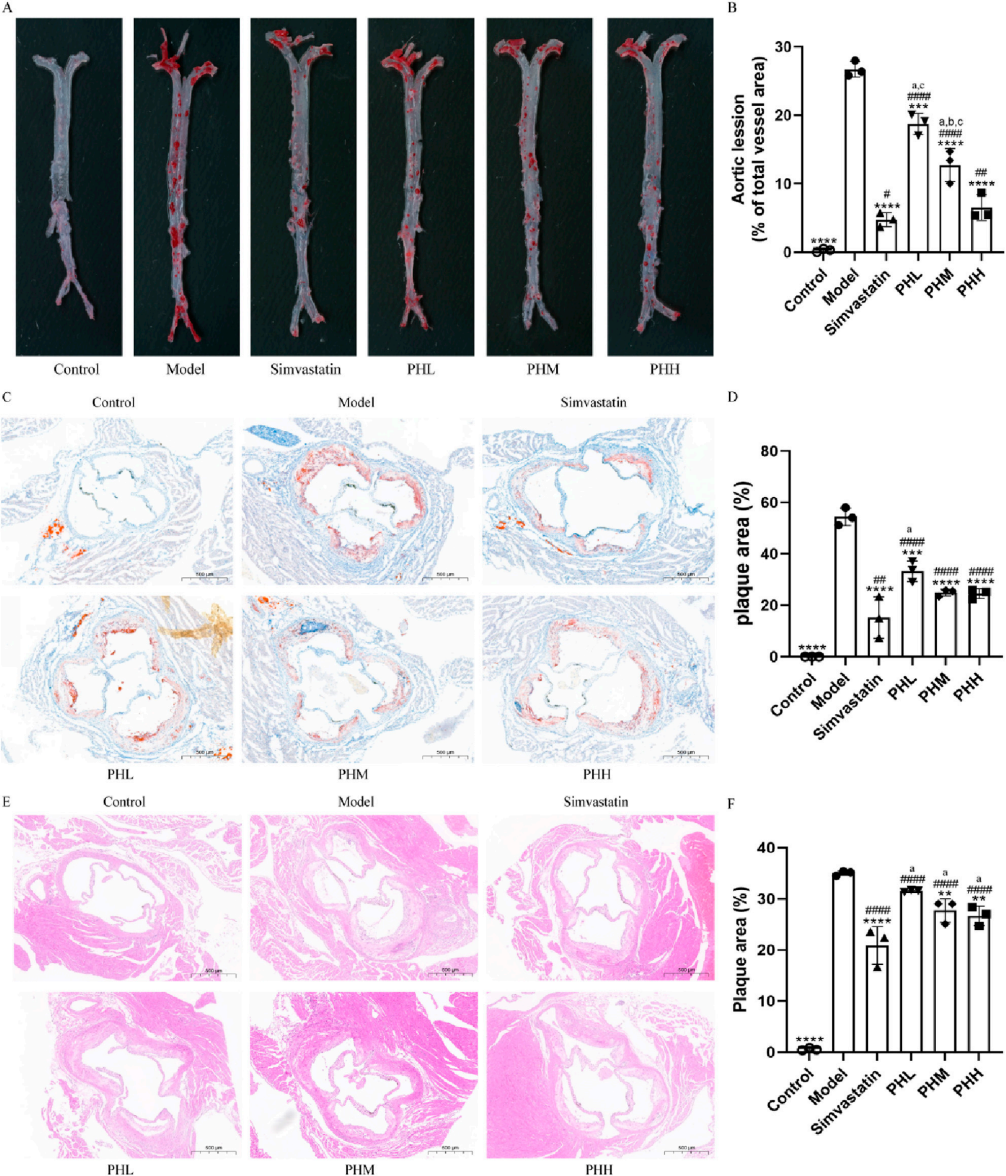
PH attenuated atherosclerosis injury in HFD-fed ApoE^−/−^ mice. **(A, B)** Image of enface aortas stained with ORO, and quantitative analysis of the aortic lesion ratio. **(C, D)** Image of aortic roots sections stained with ORO, and quantitative analysis of the aortic roots cross-sectional lesions ratio. **(E, F)** Image and quantitative analysis of aortic roots sections stained with H&E, illustrating the proportion of the cross-sectional lesions in the aortic roots. All data are shown as the mean ± SD (n = 3). **P < 0.01, ***P < 0.001, ****P < 0.0001, vs. Model; ^#^ *P* < 0.05, ^##^ *P* < 0.01, ^####^ *P* < 0.0001, vs. Control; ^a^ *P* < 0.05 vs. Simvastatin; ^b^ *P* < 0.05 vs. PHL; ^C^ *P* < 0.05 vs. PHH.

Atherosclerotic plaque size was measured to assess the pathological changes in atherosclerosis. The ratio of the plaque area to the entire aorta or aortic root in the model group increased after HFD feeding. The results of ORO staining revealed that the rising trend in atherosclerotic plaque area was inhibited, and even reduced in different doses of the PH or simvastatin groups as compared with the model group (Figures 2A, C). The aorta inner wall was smooth without any evidence of obvious plaques attached in the control group, whereas lipid deposition was more prominent with an amount of foam cells within the plaque and aortic vessel stenosis in the model group (Figures 2A, C). Specifically, quantitative analysis revealed that the proportion of aortic lesions to the total aortic vessel area in the PH group showed a descending trend compared to that of the model group (*P* < 0.0001) (Figure 2B). The therapeutic effects of PHM and PHH on atherosclerosis were significant. Consistently, the proportion of cross-sectional lesions of the aortic roots reduced in the PHM and PHH groups, compared to the model group (*P* < 0.01) (Figure 2D). The results of H&E staining also suggested that the atherosclerotic plaques and lesions in the Model group were higher than those in the Control group (Figure 2E). The lesion area in the aortas in the PH and simvastatin groups mice was significantly reduced (*P* < 0.001) (Figure 2F). In summary, PHM, PHH, and simvastatin had exerted significant effects on atherosclerosis and delayed plaque deterioration.

### 3.3 PH improved inflammatory cytokine levels in HFD-fed ApoE^−/−^ mice

**FIGURE 3:**
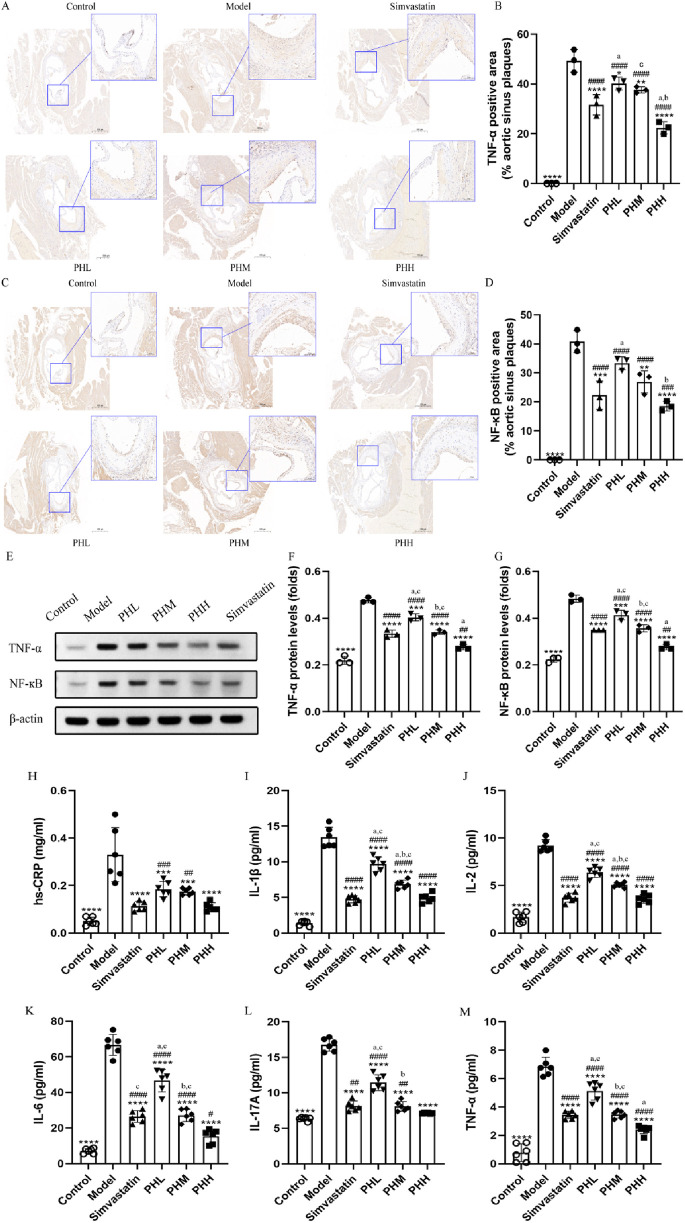
PH inhibited the activation of serum inflammatory cytokine in HFD-fed ApoE^−/−^ mice. **(A)** and **(B)** Image and quantitative analysis of immunohistochemical staining of the aortic sinus and the proportion of the TNF-α positive area to the aortic sinus plaques (n = 3). **(C, D)** Image and quantitative analysis of immunohistochemical staining of the aortic sinus and the proportion of the NF-кB positive area to the aortic sinus plaques (n = 3). **(E)** Protein expression of TNF-α and NF-кB. **(F, G)** Quantitative analysis of TNF-α and NF-кB protein levels in the aortic sinus plaques (n = 3). **(H–M)** Serum hs-CRP, IL-1β, IL-2, IL-6, IL-17A, and TNF-α levels (n = 6). *P < 0.05, **P < 0.01, ***P < 0.001, ****P < 0.0001, vs. Model; ^#^ *P* < 0.05, ^##^ *P* < 0.01, ^###^ *P* < 0.001, ^####^ *P* < 0.0001, vs. Control; ^a^ *P* < 0.05 vs. Simvastatin; ^b^ *P* < 0.05 vs. PHL; ^C^ *P* < 0.05 vs. PHH.

Besides lipid abnormalities, the progression of atherosclerosis is also influenced by systemic inflammation to a certain extent. Given that inflammatory cytokines have been recognized as an index of atherosclerosis in epidemiological studies, immunohistochemical staining of aortic root plaques was conducted. We observed that the expression of TNF-α and NF-кB was more prominent in mice fed an HFD than in mice fed a chow diet. Nevertheless, dietary supplementation with PH and simvastatin diminished the intensity of the staining, implying that a strong antagonistic action was exerted against the inflammatory reaction during the process of atherosclerotic plaque formation (Figures 3A, C). Quantitative analysis was utilized to assess the specific efficacy of PH and simvastatin. As shown in Figures 3B, D, the ratio of TNF-α and NF-кB positive area to the aortic sinus plaques in PH and simvastatin groups were dramatically decreased (*P* < 0.0001), especially obvious in PHH group. Thus, the therapeutic effect of PHH in down-regulating TNF-α and improving inflammation factor levels is superior to that of simvastatin (Figure 3B). Consistently, we observed that the downregulation of NF-κB protein in aortic plaque in PHM, PHH, and simvastatin group was more significant than that in model group (*P* < 0.0001) (Figure 3E), with the lowest protein expression in the PHH group (Figures 3F, G).

Furthermore, we measured the levels of other important inflammatory mediators to confirm the effects of PH on atherosclerosis. The results were consistent with the previously reported changes, demonstrating that the serum levels of hs-CRP, TNF-α, IL-1β, IL-2, IL-6, and IL-17A in model group mice dramatically increased in mice fed an HFD (Figures 3H–M). After PH and simvastatin administration, a dose-dependent decrease in the inflammatory cytokine levels was observed, with the lowest level in PHH group (*P* < 0.05) (Figure 3M). This suggested that inflammatory cytokines, represented by TNF-α and NF-кB, are closely related to the progression of atherosclerotic plaques. PH reduces inflammatory factor levels, thereby improving atherosclerotic plaque formation, with the most pronounced effects found in the PHH group.

### 3.4 PH inhibited the enhancement of TMA and TMAO level in HFD-fed ApoE^−/−^ mice

**FIGURE 4:**
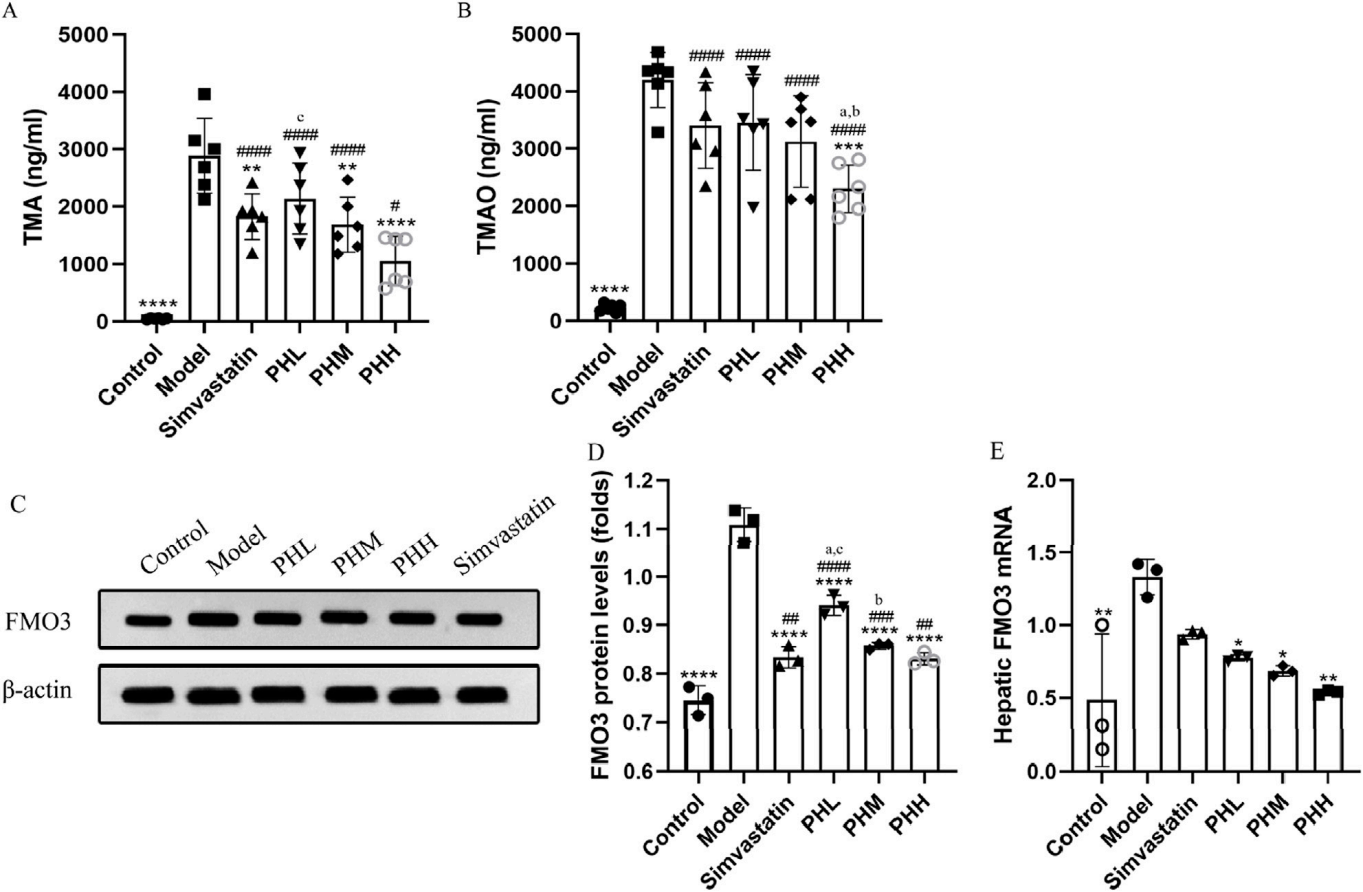
PH regulated TMA/TMAO metabolism involving hepatic FMO3 in HFD-fed ApoE^−/−^ mice. **(A, B)** Serum TMA and TMAO levels (n = 6). **(C, D)** Western blot detection and quantitative analysis of FMO3 protein in liver tissues expression (n = 3). **(E)** mRNA levels of FMO3 in liver tissues quantified by qPCR (n = 3). All data are shown as the mean ± SD. *P < 0.05, **P < 0.01, ***P < 0.001, ****P < 0.0001, vs. Model; ^#^ *P* < 0.05, ^##^ *P* < 0.01, ^###^ *P* < 0.001, ^####^ *P* < 0.0001, vs. Control; ^a^ *P* < 0.05 vs. Simvastatin; ^b^ *P* < 0.05 vs. PHL; ^C^ *P* < 0.05 vs. PHH.

To investigate the underlying mechanism of PH on atherosclerosis, atherosclerotic markers TMAO, and the TMAO metabolic pathway-related proteins were assessed. We observed that the HFD containing 1% choline induced an elevation of TMA and TMAO levels in the model group mice. Similar to the findings of previous studies (Li et al., 2021), choline accelerated atherosclerotic progression in the present study. We observed the attenuation of TMA and TMAO in the PHH group, compared to the model group (*P* < 0.001). Notably, TMA and TMAO levels were reversed by PH supplementation at high doses, indicating that the therapeutic effect of PHH was more prominent than that of the others (Figures 4A, B).

Hepatic FMO3 is a crucial enzyme involved in the conversion of TMA to TMAO. Thus, we focused on assessing the dynamic changes in FMO3 expression and activity. In line with previous findings, the results demonstrated that compared to the control group, the protein and mRNA levels of FMO3 in mice were upregulated in the model group (*P* < 0.01). PH downregulated the protein and mRNA expression of FMO3, which was more evident in terms of protein expression of FMO3. Similarly, a high dose of PH exhibited an unexpected effect on TMA/TMAO metabolism (Figures 4C–E). In conclusion, we speculated that activation of the TMA/FMO3/TMAO pathway is an important incentive for atherosclerosis, and PH inhibits TMAO generation through the involvement of hepatic FMO3.

### 3.5 The effect of PH on microbial diversity and distribution during the process of atherosclerosis

**FIGURE 5:**
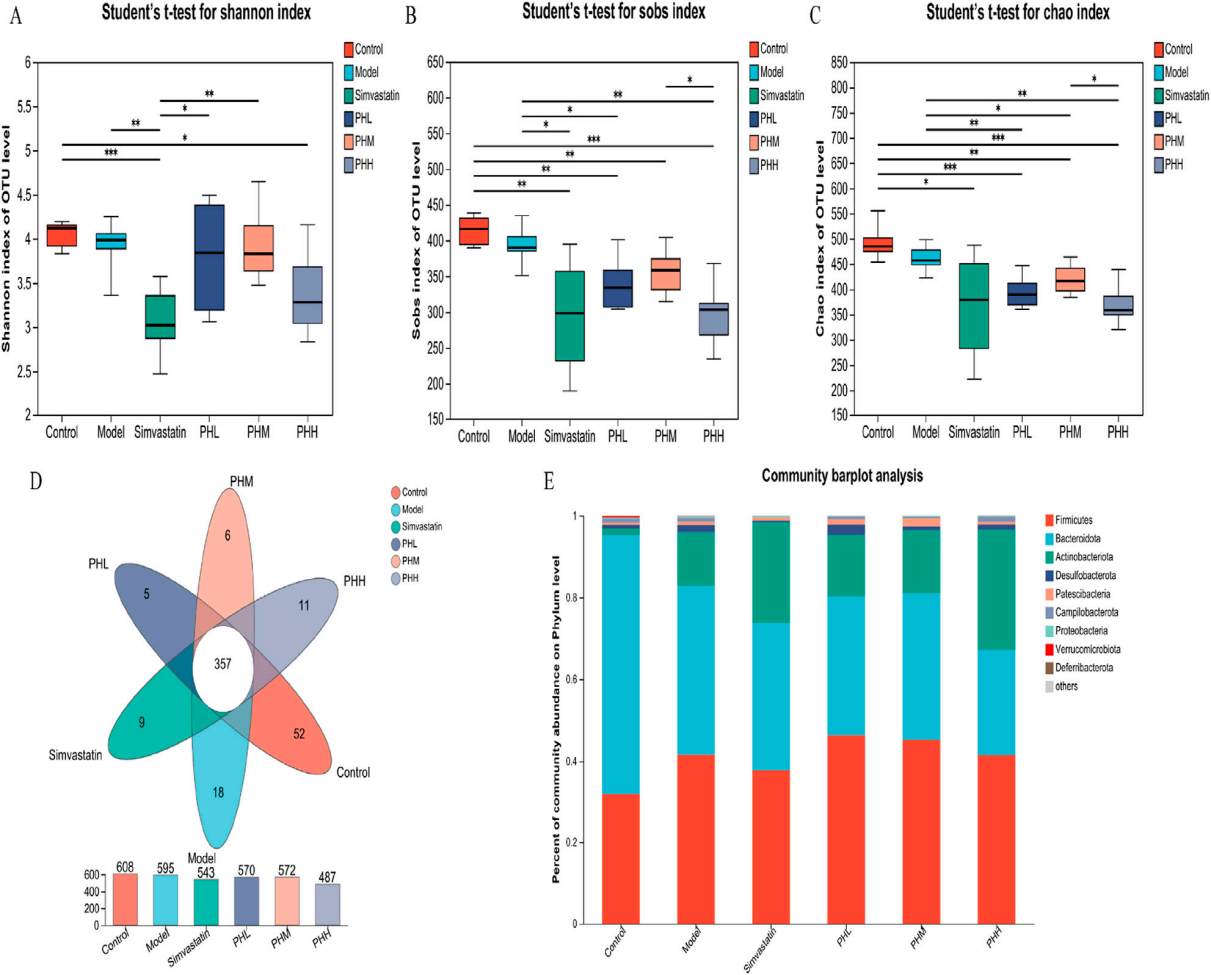
**(A–C)** Alpha diversity was analyzed by the Shannon, Sobs, Chao index; **(D)** Venn diagrams of OTUs; **(E)** Taxonomic composition/microbial compositions at the phylum level. *P < 0.05, **P < 0.01, ***P < 0.001, ****P < 0.0001.

To explore the microbial mechanism, 16S rRNA gene sequencing with a 97% similarity criterion for operational taxa was acquired from 36 samples. After sequence quality control and splicing, 3375 operational taxonomic units (OTUs) were identified. The rank-abundance curve declined smoothly and eventually flattened (Supplementary Figure S1A). The species cumulative and rarefaction curve revealed that the sequences per sample were sufficient and the sequencing depth was qualified for analysis later (Supplementary Figures S1B, C). Alpha (α) diversity analysis revealed that microbial diversity and abundance were reduced after an HFD intervention (Supplementary Figures S2, S3; Supplementary Tables S5, S6; Figures 5A–C); however, this trend was reversed or attenuated after PH administration (Figures 5D, E; Supplementary Figure S7). The gut microbiota characteristics were explored and analyzed at different levels, and 11 phyla, 14 classes, 32 orders, 50 families, 116 genera, and 201 species were identified. Moreover, the detail of distribution of different species were shown in Supplementary Figures S4–S6. At the phylum level, the proportion of *Desulfobacterota* markedly increased in the model group, while decreased in the PHM, PHH and simvastatin groups (Figure 5E).

### 3.6 The improvement of HFD-fed induced atherosclerosis by PH may be related to the alteration of gut microbiota

**FIGURE 6:**
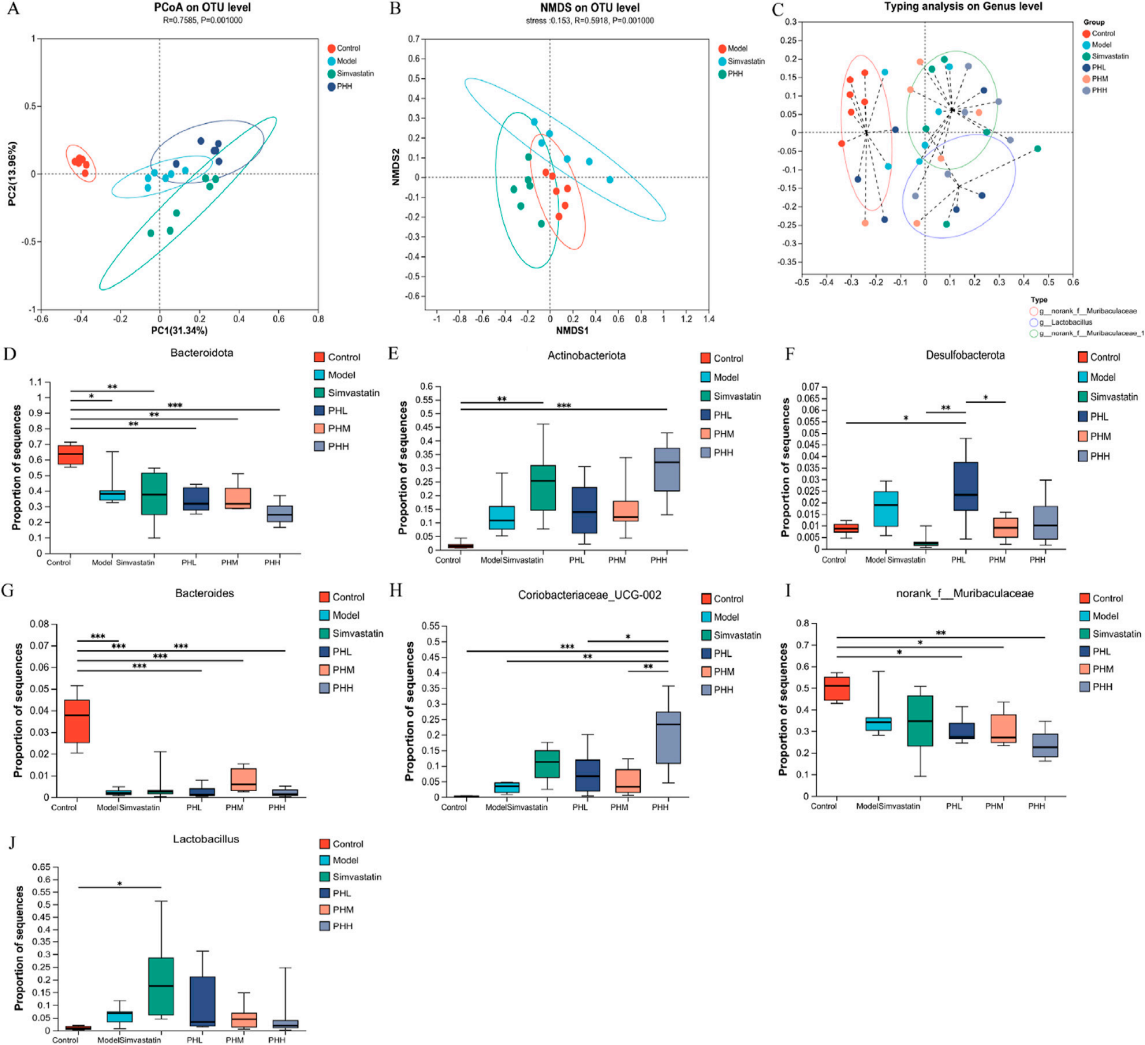
**(A)** PCoA plot; **(B)** NMDS analysis; **(C)** Effects of PH or simvastatin on enterotype of mice; **(D–F)** Multigroup difference analysis at the phylum level; **(G–J)** Multigroup difference analysis at the genus level; n = 6. *P < 0.05, **P < 0.01, ***P < 0.001.

Diversity analysis was performed to identify the alterations of gut microbial structure. The PCoA plot showed a remarkable separation among the four groups (control, model, PHH, and simvastatin) along the PC2 axis, which explained 13.96% of the variation in the microbial structure (Figure 6A). The segregated microbial structure among the three groups (control, PHH, and simvastatin) was consistently visualized using non-metric multidimensional scaling (NMDS) analysis (Figure 6B). The microbiota composition was clustered by partitioning around the medoids, and three enterotypes were identified. We identified that the proportion of *Lactobacillus* increased with the administration in PH or simvastatin (Figure 6C). Notably, the enterotypes in the PHH and simvastatin groups were mainly clustered into *g_Lactobacillus* and *g_norank_f_Muribaculaceae_1 group*, indicating that PHH and simvastatin reversed the HFD-induced enterotype switch (Supplementary Figure S8). Presumably, PHH and simvastatin protected the atherosclerotic mice against microbes. Accumulating evidence indicates that the HFD-induced decrease in gut microbial diversity can be restored to some extent by PH treatment. *Bacteroidetes* and *Bacteroides* were enriched in the control group, whereas *Alistipes*, *f_*Clostridiaceae, and *f_*Defluviitaleaceae were the predominant genera in the model group. PH administration significantly improved the proportion of *p_ Actinobacteria*, *f_Atopobiaceae*, and *g_*Coriobacteriaceae*_UCG-002*, whereas it decreased the proportions of *f_*Clostridiaceae and *f_*Defluviitaleaceae in the model group mice (Supplementary Figure S9). Finally, the multigroup difference analysis suggested that HFD increased the abundance of *Antinobacteriota*, *Desulfobacterota*, and Coriobacteriaceae*_UCG-002* in the model group and reduced the abundance of *Bacteroidetes*, *Bacteroides*, and *norank_f_Mribaculaceae*, whereas PH treatment reversed these changes (Figures 6D–J).

### 3.7 Uncovering the anti-atherosclerotic mechanism of PH: targeting the functional capacity of gut microbiota

**FIGURE 7:**
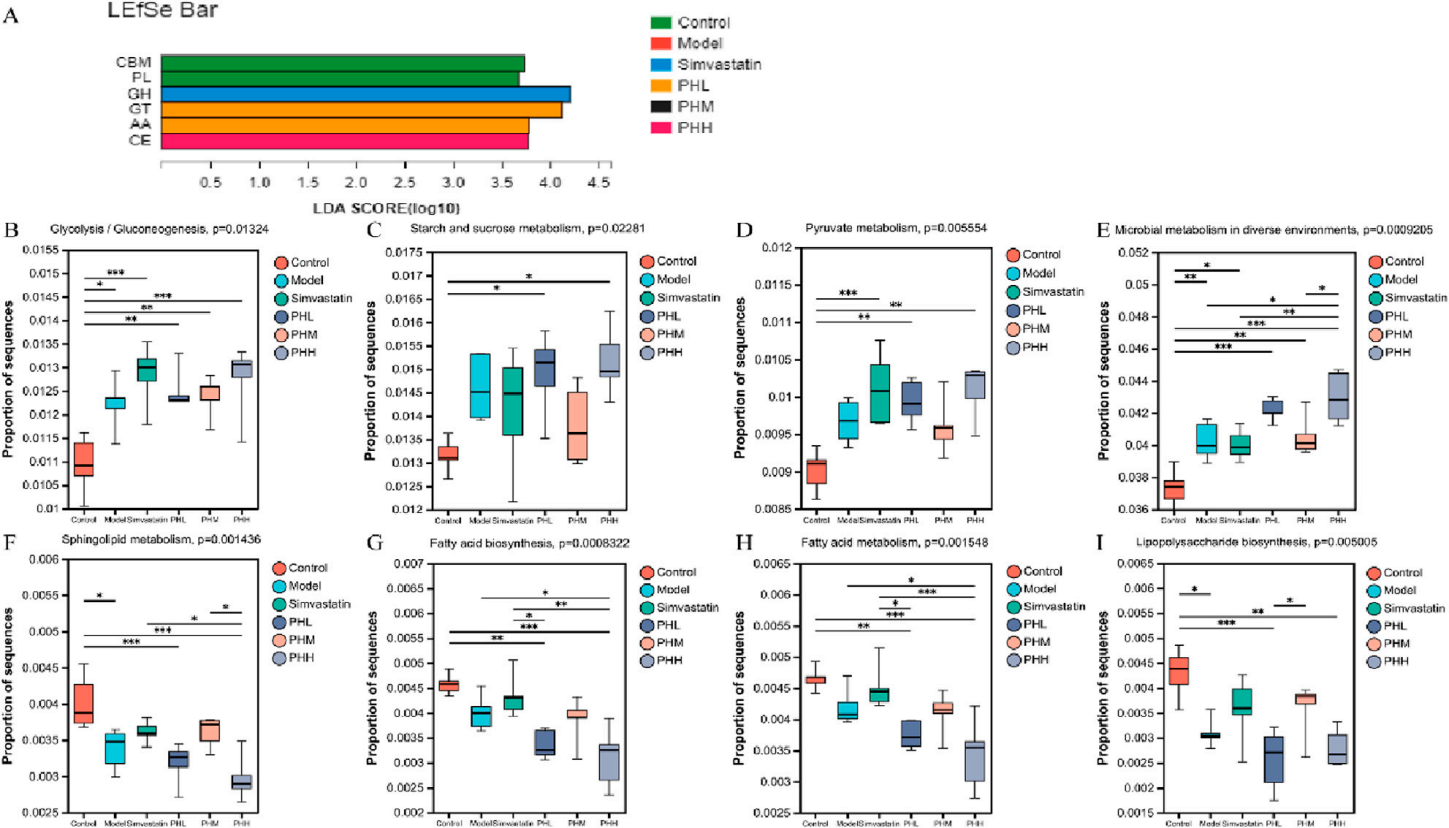
**(A)** LEfSe analysis of the differential enrichment of CAZymes at the family level (LDA>3); **(B–I)** Multigroup comparison analysis of KEGG pathways; *P < 0.05, **P < 0.01, ***P < 0.001.

We further conducted the metagenomic sequencing analysis to investigate the core functional capacity of different genes. Based on the LEfSe analysis, we observed that 37 differential KEGG pathways were identified in PHL and PHH groups, and ribosome, protein export, and RNA polymerase pathways were enriched in the model group. Most of the 37 differentially expressed functional pathways were highly enriched in the carbohydrate metabolism and membrane transport-related pathways. Among these, ABC transporters, carbon metabolism, glycerolipid metabolism, and glucagon signaling pathways were identified in the PHL group, and the biosynthesis of amino acids, starch, and sucrose metabolism were increased in the PHH group (Supplementary Figure S10A). Additionally, fatty acid metabolism, fatty acid and lipopolysaccharide biosynthesis were enriched in the control group, whereas the enrichment was reduced in the PHH group. A similar trend was observed in the multi-group comparison analysis (Figures 7B–I). Based on these results, we investigated the enrichment of CAZymes by LEfSe analysis. At the class level, glycosyl transferases (GTs) and carbohydrate esterases (CEs) were highly enriched in the PHL and PHH groups, whereas glycoside hydrolases (GHs) were mainly present in the simvastatin group (Figure 7A). The HFD-induced abundance of CAZymes was restored by PH administration, at the family level. Several CAZymes, including GT2_Glyco_tranf_2_3 and CE1, were enriched in the PHH group (Supplementary Figure S10B).

### 3.8 Association analysis among atherosclerotic parameters, microbiota species, and microbial functions

**FIGURE 8:**
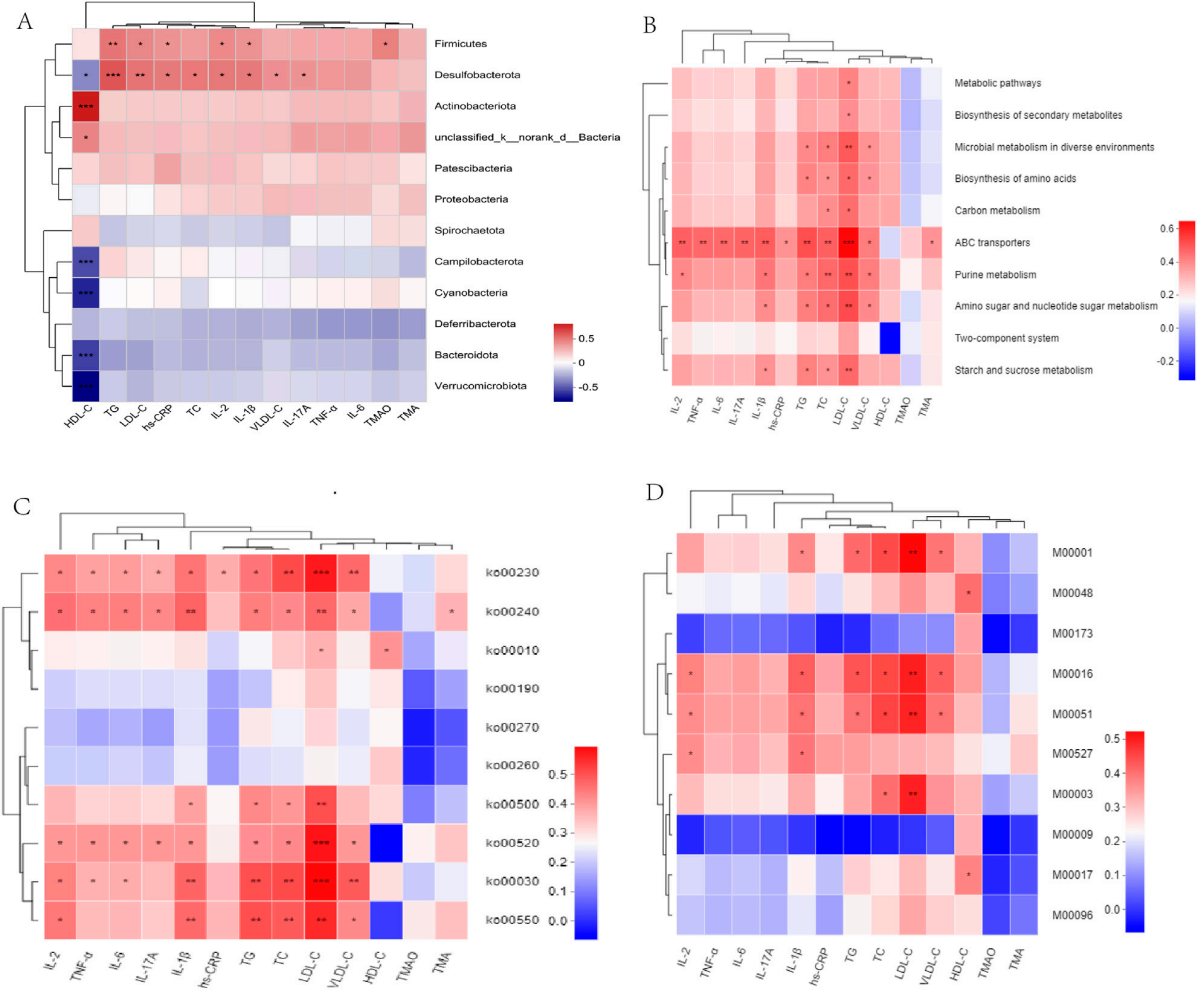
**(A)** Correlation heatmaps of gut microbiota and atherosclerotic parameters at the phylum levels. **(B–D)** Correlation between atherosclerotic parameters and KEGG functional pathways at level 3, level 2, and modules. *P < 0.05, **P < 0.01, ***P < 0.001.

We constructed a heatmap to visualize the associations between the gut microbiota and bioinformatic parameters. *Desulfobacterota* demonstrated a significant positive correlation with lipid levels (TG, TC, and LDL-C) and inflammatory cytokines (hs-CRP, IL-2, IL-1β, and IL-17A). Additionally, *Firmicutes* displayed a similar trend with TG, LDL-C, IL-2, and IL-1β (Figure 8A). Notably, *Firmicutes*, *Lachnoclostridium* and *Turicibacter* demonstrated significant positive correlations with TMA, TMAO, lipid, and inflammatory cytokine levels. *Desulfovibrio*, *Muribaculum*, and *Allobaculum* also correlated with lipid levels and inflammatory cytokines. *Bacteroides*, Prevotellaceae*_Ga6A1_group*, and *Eubacterium_fissicatena_group* negatively correlated with TMA, TMAO, lipid levels, and inflammatory cytokines (Supplementary Figure S11A), thereby indicating that these microbial biomarkers may be involved in the development of atherosclerosis.

Associations between atherosclerotic variables, the KEGG functional pathways, and gut microbiota were analyzed. At Level 3, ABC transporters demonstrated positive correlations with lipid levels (TG, TC, LDL-C, and VLDL-C) and inflammatory cytokines (IL-2, IL-6, IL-17A, IL-1β, hs-CRP, and TNF-β). In particular, LDL-C demonstrated a positive correlation with the metabolic pathways, including carbon, starch, and sucrose metabolism (Figure 8B). At level 2, ko00520, ko00030, and ko00550 demonstrated similar associations with the lipid and inflammatory cytokine levels (Figure 8C). At module level, M00001 (Glycolysis), M00016 (Lysine biosynthesis), and M00051 (*De novo* pyrimidine biosynthesis) demonstrated positive correlations with lipid and IL-1β levels (Figure 8D). *Uncultured_bacterium_g_Turicibacter*, and *uncultured_bacterium_g_Desulfovibrio* exhibited positive correlations with TMAO, lipids, and inflammatory cytokine levels, whereas *Bacteroides_acidifaciens* exhibited negative correlations with TMAO, lipid, and inflammatory cytokine levels (Supplementary Figure S10B). In addition, we observed that metabolic pathways, including starch and sucrose metabolism, and ABC transporters, including M00001, M00051, and M00003, exhibited a positive correlation with *s_Desulfovibrio_fairfieldensis* and *s_Allobaculum_stercoricanis*, but a negative correlation with *Bacteroides* spp. and *s_Lactobacillus_murinus* (Supplementary Figures S12A, B).

### 3.9 PH regulated the glycolipid metabolic pathways via TMA-producing bacteria related to atherosclerosis

**FIGURE 9:**
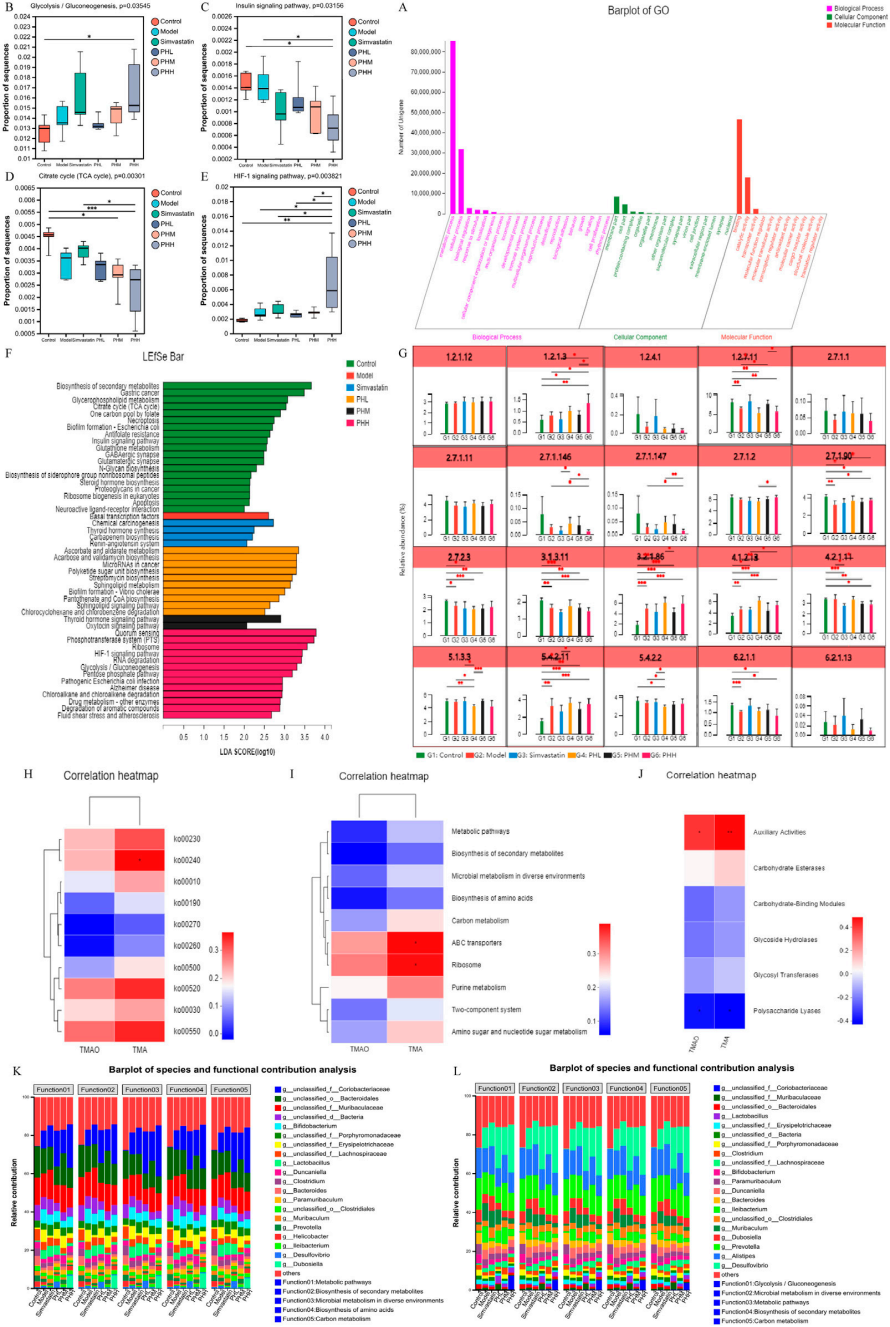
**(A)** GO function annotation analysis; **(B–E)** Multigroup variance analysis of KEGG pathways based on TMA-producing bacteria gene set; **(F)** Linear discriminant analysis of TMA-producing bacteria gene set; **(G)** Analysis of intergroup difference in metabolic pathways; **(H–J)** Spearman’s correlation analysis between metabolic pathways and TMA/TMAO; **(K, L)** Analysis of species function contribution. *P < 0.05, **P < 0.01, ***P < 0.001.

Considering the pathways involved in the choline metabolism, TMAO, and associated gut microbiota, TMA-producing bacteria were selected for gene set construction. The gene set included CutC-related bacteria, which may have contributed to the therapeutic effects of PH. Critical KEGG pathways among different groups were identified using linear discriminant analysis. Glycolysis/gluconeogenesis, fluid shear stress, atherosclerosis, drug metabolism, and the HIF-1signaling pathway were highly enriched in the PHH group (Figure 9F; Supplementary Figure S13). Further multigroup comparison analysis revealed that the abundance of glycolysis/gluconeogenesis and HIF-1signaling pathway markedly increased after PHH treatment (Figures 9B–E). Thus, an analysis of intergroup differences in metabolic pathways was conducted based on these findings. The abundance of 6-phospho-beta-glucosidase [EC:3.2.1.86], which is involved in the glycolysis/gluconeogenesis metabolic pathway. The abundance of phosphoglycerate mutase [EC:5.4.2.11] and fructose-bisphosphate aldolase [EC:4.1.2.13] were increased in the model group. In addition, other related enzymes, such as 6-phosphofructokinase [EC:2.7.1.90] and fructose-bisphosphatase [EC:3.1.3.11], may also exerted an important effect (Figure 9G). We hypothesized that phosphoglycerate mutase and fructose-bisphosphate aldolase were the main contributors of glycolysis/gluconeogenesis pathway. Moreover, the gene ontology (GO) functional annotation results suggested that the biological processes (BP) of each group were mainly enriched in metabolic and biosynthetic processes (Figure 9A).

Correlation analyses between metabolic pathways and TMA/TMAO revealed that ko00240, ABC transporters, and auxiliary activities positively correlated with TMA, suggesting that TMA-producing bacteria were mainly involved in the progression of atherosclerosis via metabolic pathways and related enzymes (Figures 9H–J). Moreover, we identified the top 20 species that contributed to the differences in pathways at level 3. Metabolic functions are involved in different pathways, including glycolysis/gluconeogenesis, microbial metabolism, biosynthesis of secondary metabolites, and carbon metabolism. *Desulfovibrio*, *Prevotella*, *Muribaculum*, *Clostridium*, *unclassified_f_*Lachnospiraceae, *unclassified_o_Bacteroidales*, *unclassified_f_Muribaculaceae*, and *unclassified_f_*Coriobacteriaceae were contributed to these metabolic pathways (Figures 9K, L).

**FIGURE 10:**
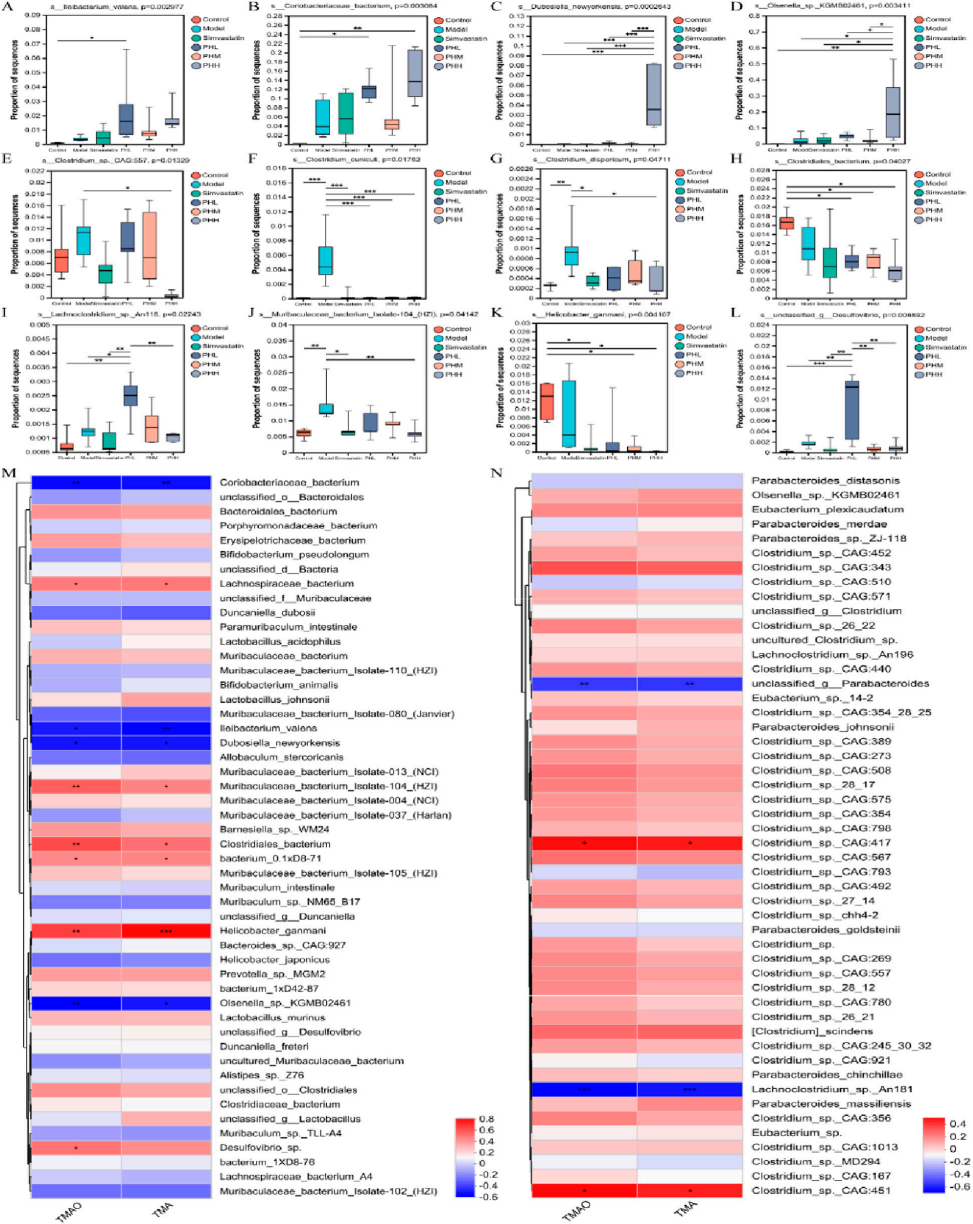
**(A–L)** Multigroup variance analysis of TMA-producing bacteria gene set; **(M, N)** Spearman’s correlation analysis between TMA-producing bacteria and TMA/TMAO. *P < 0.05, **P < 0.01, ***P < 0.001.

Finally, we further investigated the association between the TMA-producing bacteria and TMA/TMAO levels. Interestingly, Lachnospiraceae*_bacterium*, *Muribaculaceae_bacterium*_Isolate-104_(HZI), *Clostridiales_bacterium*, bacterium_0.1xD8-71, *Helicobacter_ganmani*, *Desulfovibrio_*sp., *Clostridium*_sp._CAG:417, and *Clostridium*_sp._CAG:451 exhibited positive correlations with TMA and TMAO. However, Coriobacteriaceae*_bacterium*, *Ileibacterium_valens*, *Dubosiella_newyorkensis*, *Olsenella*_sp._KGMB02461, *unclassified_g_Parabacteroides*, and *Lachnoclostridium*_sp. An181 expression exhibited negative correlations with TMA and TMAO (Figures 10M, N). Specifically, the abundance of *s_Clostridium*_sp._CAG:557, *s_Clostridium_cuniculi*, and *s_Clostridium_disporicum* increased in the model group, while the HFD-induced increase in the abundance of *Clostridium* spp. Was partially restored by PH administration. In addition, similar changes were observed in certain gut microbial species, such as *s_Lachnoclostridium*_sp._An118, *s_Muribaculaceae_bacterium*_Isolate-104_(HZI), and *s_unclassidied_g_Desulfobibrio* (Figures 10A–L). We speculated that CutC-related bacteria, such as Lachnospiraceae, *Muribaculaceae*, *Clostridium*, and *Desulfovibrio* prompted the progression of atherosclerosis via glycolipid metabolic pathways.

## 4 Discussion

In this study, we uncovered the anti-atherosclerotic mechanism of PH through gut microbiota and metabolites. Our results suggested that PH could regulate the glucolipid metabolism-related pathway, attenuate inflammatory cytokine levels, and reduce atherosclerotic plaques by remodeling gut microbiota. Modern pharmacological studies have shown that polydatin is extensively used for the treatment of atherosclerosis and systemic metabolic disorders owing to its medicinal properties, including anti-inflammatory (Tang et al., 2018), antioxidant (Lv et al., 2019), and lipid metabolism modulation (Huang et al., 2018). Furthermore, hawthorn flavonoids alleviate metabolic hyperlipidemia by altering bacterial and gut-derived metabolites related to cholesterol homeostasis (Hu et al., 2022). Dyslipidemia plays an important role in the development of ASCVD (Ballard-Hernandez and Sall, 2023). Statins reduce LDL cholesterol levels and prevent ASCVD by inhibiting 3-hydroxy-3-methylglutaryl-CoA reductase (HMGCR) and increasing the expression of low density lipoprotein receptor (LDLR) (Grundy et al., 2018). Proprotein convertase subtilisin/kexin type 9 (PCSK9) is a serine protease that homologous binds with epidermal growth factor precursors to the LDLR domain A (EGF-A) in the liver and initiates degradation of LDLR via the lysosomal pathway, resulting in elevated plasma LDL-C levels (Hao et al., 2022). Blocking the direct interaction between PCSK9 and LDLR may be the key point to ameliorating hyperlipidemia and ASCVD (Wang et al., 2024).

Our clinical trial confirmed that polydatin combined with hawthorn flavonoids can exert anti-atherosclerotic effect on patients with carotid atherosclerosis by promoting plaque stability and decreasing the inflammatory factors levels (Liu et al., 2014), which suggested that the combination of polydatin and hawthorn flavonoids could exhibit an amazing anti-atherosclerotic effect. Our previous research confirmed that the mice in the model group may have experienced an increase in the serum TC, TG, and LDL-C levels; however, hawthorn flavonoids and simvastatin reversed these changes and increased HDL-C levels (Wang et al., 2019). Furthermore, polydatin moderates the lipid metabolism via inhibiting the formation of peritoneal macrophage-derived foam cells in ApoE^−/−^mice (Wu et al., 2015). It is reasonable to speculate that polydatin combined with hawthorn flavonoids may have excellent lipid-regulating effects. Our data revealed that PH could reverse dyslipidemia by reducing lipid levels (TC, TG, LDL-C, and VLDL-C) and body weight and increasing HDL-C levels.

Our previous results demonstrated that polydatin could reduce TNF-α and IL-1β levels in the peritoneal macrophages of ApoE^−/−^mice (Wu et al., 2015), and hawthorn flavonoids could inhibit the elevation of hs-CRP and IL-1β (Wang et al., 2019). Additionally, our clinical trial data confirmed that 15 g of *P. cuspidatum* combined with 10 g of hawthorn granule formula exerted cardioprotective effects by reducing hs-CRP, TNF-α, and IL-6 levels. We also observed that the plaque score decreased, and the level of plaque stability was strongly enhanced in patients with carotid atherosclerosis (Wu et al., 2021). Furthermore, our previous research revealed that hawthorn flavonoids (Wang et al., 2019) and polydatin (Zhang et al., 2023) could individually improve atherosclerotic plaques in ApoE^−/−^mice. Consistent with these findings, we observed that PH could reduce serum inflammatory factors (IL-1β, IL-2, IL-6, IL-17A, hs-CRP and TNF-α) levels and improve atherosclerotic plaques.

In our study, the alpha diversity analysis revealed that *Firmicutes*, *Bacteroidetes*, *Actinobacteria*, and *Desulfobacterota* accounted for the majority of species in our study, and the microbial diversity and abundance were reduced after the HFD intervention. *Firmicutes* and *Bacteroidetes*, as dominant bacteria in a healthy body, are involved in lipid regulation and BAs metabolism. *Firmicutes* may increase the formation of lipopolysaccharides and other metabolic endotoxins leading to chronic inflammation, while *Bacteroidota*, as a Gram-negative anaerobic bacterium, plays an important role in maintaining immune homeostasis and regulating immunity (The Human Microbiome Project Consortium, 2012). Our result was similar to previous studies, with *Bacteroidetes* and *Firmicutes* constituting more than other species, with *Actinobacteria*, *Desulfobacterota* followed them. Although the species abundance or number of the microbiota varied between individuals, the gut microbiota was semblable among individuals at higher taxonomic levels, such as the phylum level. In particular, we observed that the proportion of *Desulfobacterota* was increased in the model group, however, it was decreased in the PHM, PHH and simvastatin groups. *Desulfovibrio*, a Gram-negative bacterium, is involved in lipid metabolism and inflammation by producing lipopolysaccharide to trigger the release of IL-1β, IL-6, and TNF-β and alter intestinal permeability and microbial composition (Zhang et al., 2018). Previous research revealed that HFD-induced gut microbial alterations in atherosclerotic mice are mainly reflected in the enrichment of *Desulfovibrio* (Zhu et al., 2018b). *Muribaculaceae* (S24-7) can inhibit the inflammation (Luo et al., 2021), gut microbial dysbiosis, and intestinal dysfunction by degrading mucin (Lee et al., 2019). Simvastatin attenuates HFD-induced hyperlipidemia by increasing the abundance of *Lactobacillus* and reducing the level of TG and TC (Zhang et al., 2020). Most studies have confirmed that *Lactobacillus* spp. can moderate lipid metabolism (Yoo et al., 2013) and reduce cholesterol availability (Pereira and Gibson, 2002). Our findings revealed that *norank_f_Muribaculaceae* was enriched in the PHM and simvastatin groups. Additionally, *Lactobacillus* was mainly increased in the simvastatin and PHL groups. Furthermore, PHH and simvastatin administration also switched the enterotype to *g_Lactobacillus* and *g_norank_f_Muribaculaceae_1* to optimize the gut microbiota composition (Watanabe et al., 2021). *In vivo* studies have revealed that the inhibition of C-C chemokine motif ligands can reduce TMAO levels and the abundance of *Muribaculaceae* to improve glucose and lipid metabolism disorders (Chang and Chen, 2021). Coriobacteriaceae*_UCG-002* belongs to the *Coribacteriaceae* family, which accelerates cholesterol absorption and exhibits a positive correlation with the levels of TG (Han et al., 2022). However, a recent report had suggested that *Coriobacteriaea*_UCG-002 can improve obesity (Jiao et al., 2023). The β-diversity analysis in our study revealed that PHH and simvastatin improved the abundance of *Actinobacteria* and *Coriobacteriaea*_UCG-002. Furthermore, harmful bacteria such as *Alistipes*, *f_*Clostridiaceae, and *f_*Defluviitaleaceae were the predominant genera in the model group. *Alistipes* have been recognized as an inflammatory indicator in most studies (Dong et al., 2019). The class *Clostridia*, which includes *f_*Clostridiaceae and *f_*Defluviitaleaceae, has often been reported to prompt the progression of colitis in mice (Zha et al., 2020), which is consistent with our observations. In our study, *Desulfobacterota*, *Firmicutes*, *Desulfovibrio*, and *Muribaculum* have strong correlations with lipid and inflammatory cytokine levels.

The KEGG functional analysis revealed increased enrichment of glycerolipid metabolism and glucagon signaling pathways in the PHL group; additionally, biosynthesis of amino acids, starch, and sucrose metabolism demonstrated increased enrichment in the PHH group. Accordingly, ko00520, ko00030, and ko00550 were positively associated with lipid and inflammatory cytokine levels at Level 2 while *s_Desulfovibrio_fairfieldensis*, *s_Allobaculum_stercoricanis* exhibited positive correlation with the metabolic pathways and starch and sucrose metabolism. A decrease in *Desulfovibrio* may contribute to the improvement of glycolipid metabolism (Huang et al., 2022) and inflammation (Shenghua et al., 2020). Thus, the microbial therapeutic effects of PH administration may be attributed to glycerolipid metabolism. Accumulating evidences reveal that gut microbiota and the metabolite TMAO may mechanistically participated in the pathogenesis of atherosclerosis (Díez-Ricote et al., 2022), although the specific mechanism has not yet been fully elucidated. Consistent with previous reports, our results confirmed that an HFD containing 1% choline increased TMA and TMAO levels in the model group mice (Thomas and Fernandez, 2021). Interestingly, we observed that PH moderated the TMA/FMO3/TMAO pathway by downregulating the protein and mRNA expression of FMO3, which is consistent with previous findings. Accumulating evidence has documented that (Lu et al., 2023), cholesterol homeostasis serves as a crucial mediator of metabolic disorders. TMAO promotes the transformation of macrophages into foam cells via cholesterol accumulation involved in the scavenger receptors SR-AI and CD36 (Wang et al., 2011). Furthermore, HFD may cause changes in BAs signaling by affecting the microbiota. BAs binds to receptors such as FXR and TGR5 and regulates host metabolism either by directly binding to promoter regions or by releasing potent regulatory factors such as fibroblast growth factor 15/19 (FGF19) in humans or pigs and glucagon-like peptide-1 (GLP-1) (Perino and Schoonjans, 2022). Previous studies have shown that the intake of a high-fat diet may cause liver damage while altering BAs and gut microbiota (Huang et al., 2024). Consistent with this mechanism, our correlation analysis revealed a positive correlation between TMA and ko00240, ABC transporters, and auxiliary activities Expression of hepatic Abcg5/g8 expression have been suggested to increase TMAO levels (Chen et al., 2019). In addition, our data suggested that the abundance of *Firmicutes*, *Turicibacter*, and *Lachnoclostridium* might participate in the process of accelerating the TMAO levels. Consistently, *Turicibacter* and *Lachnoclostridium* have been associated with butyric acid, which may promote imbalanced lipid metabolism and elevation of TMAO levels (Li et al., 2019). At the species level, we observed positive correlations of *uncultured_bacterium_g_Desulfovibrio*; *s_Desulfovibrio_fairfieldensis*; metabolic pathways, including starch and sucrose metabolism; ABC transporters, including M00001, M00051, and M00003, with *s_Allobaculum_stercoricanis*, but a negative correlation with beneficial bacteria, including *Bacteroides* spp., *s_Lactobacillus_murinus* and *s_Muribaculaceae_bacterium*. In line with previous findings, the observed alterations in the abundance of *Alloprevotella*, and *norank_f_*Desulfovibrionaceae could have contributed to an immunoprotective effect on ABC transporters, carbohydrate digestion and absorption, and glycerophospholipid metabolism pathways (Sun et al., 2022).

Since TMA-producing bacteria are the main contributors to the production of TMAO and atherosclerosis, they were identified for gene set construction to further investigate the relationship between TMAO and atherosclerosis. Glycolysis/gluconeogenesis, drug metabolism, and HIF-1signaling pathway were highly enriched in the PHH group, suggesting that a high dose of PH could change the abundance of TMA-producing bacteria and regulate the above functional pathways to attenuate the pathological process of atherosclerosis. In addition, we observed that phosphoglycerate mutase [EC:5.4.2.11] and fructose-bisphosphate aldolase [EC:4.1.2.13] were increased in mice fed an HFD, whereas PH administration could reverse these changes. Consistent with previous research, aloe polysaccharides may produce health benefits by upregulating the gene expression of fructose-bisphosphate aldolase (Liu et al., 2021). The cell-free extract of *Clostridium sporogenes* exhibited a high fructose-bisphosphate aldolase activity (Golovchenko et al., 1983). GO functional annotation also suggested that the BP of each group were mainly focused on metabolism-related processes. According to the results of the species contribution analysis, *Clostridium* was among the top five contributors to metabolic pathways, and *Desulfovibrio*, *Prevotella*, *Muribaculum*, *unclassified_f_*Lachnospiraceae, *unclassified_o_Bacteroidales* were other important species involved in metabolic processes. The genera *Clostridium* (Clostridiaceae) and *Collinsella* (Coriobacteriaceae) possess TMA lyase activity and influence the level of TMAO (Cho et al., 2017). *Clostridium*, *Desulfovibrio*, and *Collinsella* participate widely in the synthesis of TMA, which is generated from choline in dairy diets (Onyszkiewicz et al., 2020). In addition, Lachnospiraceae*_bacterium*, *Muribaculaceae_bacterium_Isolate-104_(HZI)*, *Clostridiales_bacterium*, *Desulfovibrio*_sp., *Clostridium*_sp._CAG:417, and *Clostridium*_sp._CAG:451 exhibited positive correlations with TMA and TMAO levels. The abundance of *s_Clostridium*_sp._CAG:557, *s_Clostridium_cuniculi*, and *s_Clostridium_disporicum* increased in the model group, and the PH administration partially restored the HFD-induced abundance of these *Clostridium*_spp.

## 5 Conclusion

**FIGURE 11:**
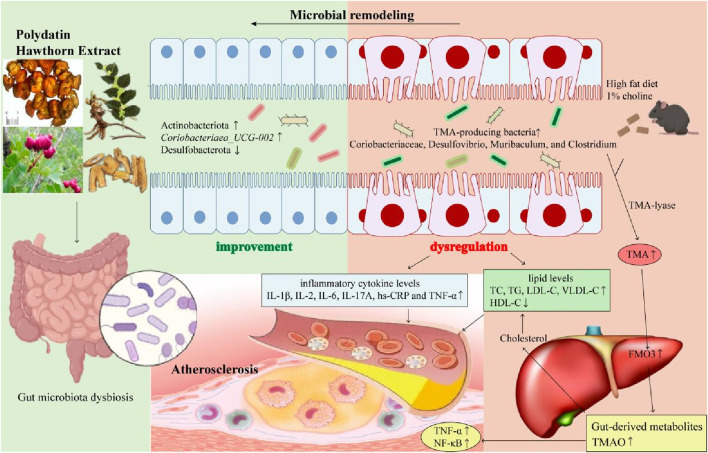
The gut microbial mechanism of polydatin combined with hawthorn flavonoids against AS. Polydatin combined with hawthorn flavonoids protects against atherosclerosis by microbial remodeling *in vivo*: 1) PH administration could increase the abundance of Actinobacteriota and *Coriobacteriaea_UCG-002*, decrease the abundance of *Desulfobacterota*; 2) HFD-induced changes in TMA-producing bacteria (Coriobacteriaceae, *Desulfovibrio*, *Muribaculum*, and *Clostridium*) increased TMA levels by TMA-lyase, upregulated the expression of FMO3, increased the gut-derived metabolites TMAO levels, which was accompanied by an increase in lipid levels and inflammatory cytokines levels. 3) PH alleviate atherosclerotic plagues by moderating gut microbiota dysbiosis to regulate the TMA/FMO3/TMAO pathway.

In conclusion, our results suggested that PH administration could reduce inflammatory factors and lipid levels, thereby attenuating the development of atherosclerotic plaques in HFD-induced mice. In addition, PH exerts its beneficial effects by remodeling the gut microbiota and regulating its derived metabolite, TMAO. In particular, PH-induced changes in TMA-producing bacteria and enzymes involved in the glycolipid metabolic pathways have important effects on atherosclerosis therapy. In this study, we found that of all treatment groups, PHH group was the most effective in improving gut microbial diversity, anti-inflammatory effects, antioxidant capacity, and lipid metabolism in a dose-dependent manner. Simvastatin group may play an anti-atherosclerosis role mainly by increasing the abundance of *Lactobacillus* and *norank_f_Muribaculaceae*. PHL and PHM may exert therapeutic effects by increasing the abundance of *Lactobacillus* and *norank_f_Muribaculaceae*, respectively. PHH and simvastatin improved the abundance of *Actinobacteria* and *Coriobacteriaea_UCG-002*. Furthermore, PHH could change the abundance of TMA-producing bacteria and regulate the Glycolysis/gluconeogenesis, drug metabolism, and HIF-1signaling functional pathways to attenuate the pathological process of atherosclerosis. To summarize, PH has the potential to regulate the abundance of TMA-producing bacteria (Coriobacteriaceae, *Desulfovibrio*, *Muribaculum*, and *Clostridium*), moderate the TMA-FMO3-TMAO pathway, and influence the related enzymes in glycolipid metabolic pathways, thereby exerting an important effect on the expression of TMAO, lipids, and inflammation, ultimately alleviating the progression of atherosclerosis (Figure 11). Nevertheless, the underlying mechanisms and new targets of gut microbial biomarkers require further investigation.

## Data Availability

The data presented in the study are deposited in the NCBI Sequence Read Archive (SRA) repository, accession number PRJNA1122934.

## Glossary

PH: polydatin and hawthorn leaf flavonoids
*P. cuspidatum*: *Polygonum cuspidatum Sieb. et Zucc*
*C. pinnatifida*: *Crataegus pinnatifida Bunge*
CVDs: Cardiovascular diseases
TMAO: Trimethylamine N-oxide
TMA: trimethylamine
FMO3: flavin-containing enzyme monooxygenase 3
HFD: high-fat diet
H&E: hematoxylin-eosin
TC: cholesterol
TG: triglyceride
LDL-C: low-density lipoprotein cholesterol
VLDL-C: very low density lipoprotein cholesterol
HDL-C: high-density lipoprotein cholesterol
NF-кB: nuclear factor-кB
TNF-α: tumor necrosis factor alpha
IL-1β: interleukin-1-beta
IL-2: interleukin-2
IL-6: interleukin-6
IL-17A: interleukin-17
OTUs: operational taxonomic units
ORFs: open reading frames
PCoA: principle coordinate analysis
NMDS: non-metric multidimensional scaling
LDA: linear discriminant analysis
LEfSe: linear discriminant analysis effect size
CAZymes: carbohydrate-active enzymes
GTs: glycosyl transferases
CEs: carbohydrate esterases
GHs: glycoside hydrolases
BP: biological processes.
