# Supplementary material for: Polydatin combined with hawthorn flavonoids alleviate high fat diet induced atherosclerosis by remodeling the gut microbiota and glycolipid metabolism
Source: Front Pharmacol. 2025 Mar 3;16:1515485. doi: 10.3389/fphar.2025.1515485 (PMC11911193; doi:10.3389/fphar.2025.1515485)
Supplement: Supplementary file 1 [file DataSheet1.pdf]

Polydatin and hawthorn extract alleviates high fat diet induced atherosclerosis by remodeling the gut microbiota and glycolipid metabolism

Dan Li *et.al.*

Data Supplementary

Table S1. Realtime PCR reaction condition

procedure	temperature	time	cycle number
predegeneration	95℃	5 min	1
denaturation	95℃	10 sec	
anneal	58℃	20 sec	40
extend	72℃	20 sec	
	95℃	15 sec	
melting curve	60℃	60 sec	1
	95℃	15 sec	

Table S2. Primer sequences

Primer sequences		5'-3'
FMO3	F	TACTGGCAAATGGGAAGTCAC
	R	CCTTTAAAACGGTTCAGTCCT
β-actin	F	CTCCTGAGCGCAAGTACTCT
	R	TACTCCTGCTTGCTGATCCAC

Table S3. Mobile phase gradient condition

The mobile phase was water (A containing 0.1 % formic acid) and acetonitrile (B containing 5 mmol/l ammonium formate, 0.1 % formic acid, Acetonitrile: Water 95:5).

Time（min）	A%	B%
0	10	90
1	10	90
3.5	50	50
3.6	10	90
5	10	90

Table S4. Mass spectrum condition

Ion source voltage	Cone-hole voltage	Ion source temperature	Desolvation temperature	Cone-hole gas flow rate	Desolvation gas flow rate
2.0 kV	10 V	150℃	450℃	10 L/h	900L/h

Table S5. Alpha diversity index comparison among groups

Estimators	Control-Mean	Control-Sd	PHH-Mean	PHH-Sd	*P*-value	Q-value

sobs	413.83	21.451	296.67	46.638	0.0002306	0.000745
shannon	4.0461	0.16123	3.3894	0.50054	0.01206	0.02413
simpson	0.045524	0.0099807	0.081402	0.03517	0.03707	0.05146
chao	492.66	35.123	369.84	41.447	0.0002483	0.000745
shannoneven	0.67155	0.023577	0.59539	0.076927	0.04288	0.05146
simpsoneven	0.055359	0.013055	0.052466	0.032931	0.8455	0.8455

Table S6. Microbial community at OTU level

Group_lable	Species_num	ratio(%)
Control&PHH&PHL&PHM&Model&Simvastatin	357	46.48
Control only	52	6.77
PHH only	11	1.43
PHL only	5	0.65
PHM only	6	0.78

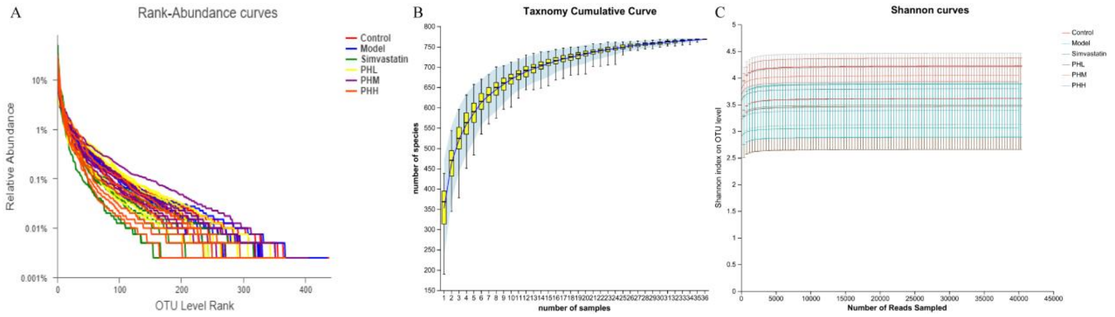

Figure S1. (A) Rank abundance curves; (B) Species accumulation curves; (C) Shannon rarefaction curve

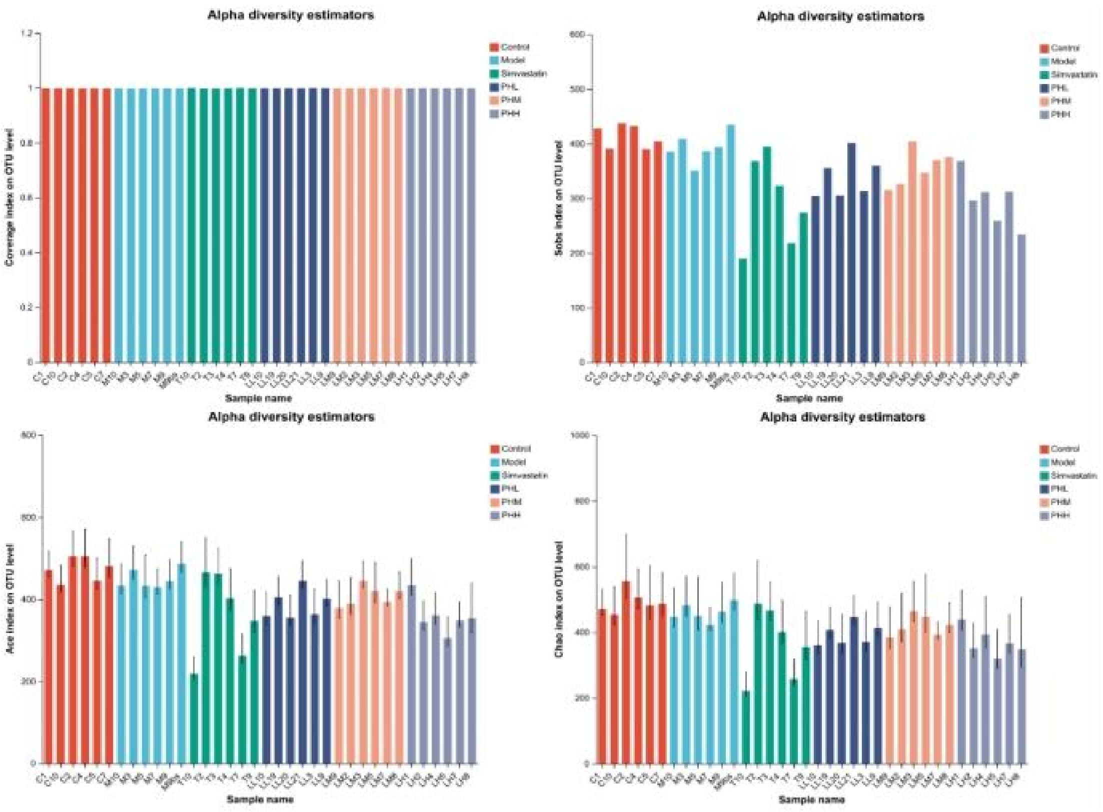

Figure S2. The distribution of coverage index and abundance index Sobs, Ace and Chao

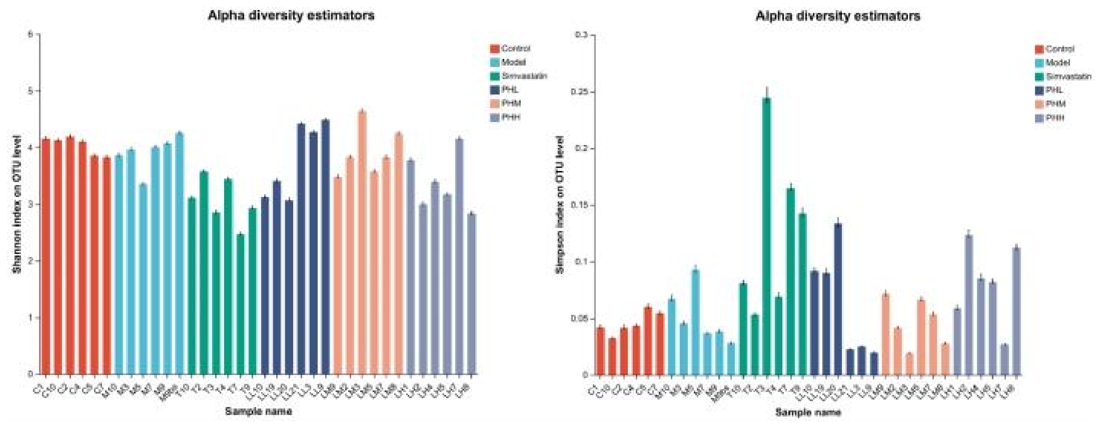

Figure S3. The distribution of diversity index Shannon and Simpson

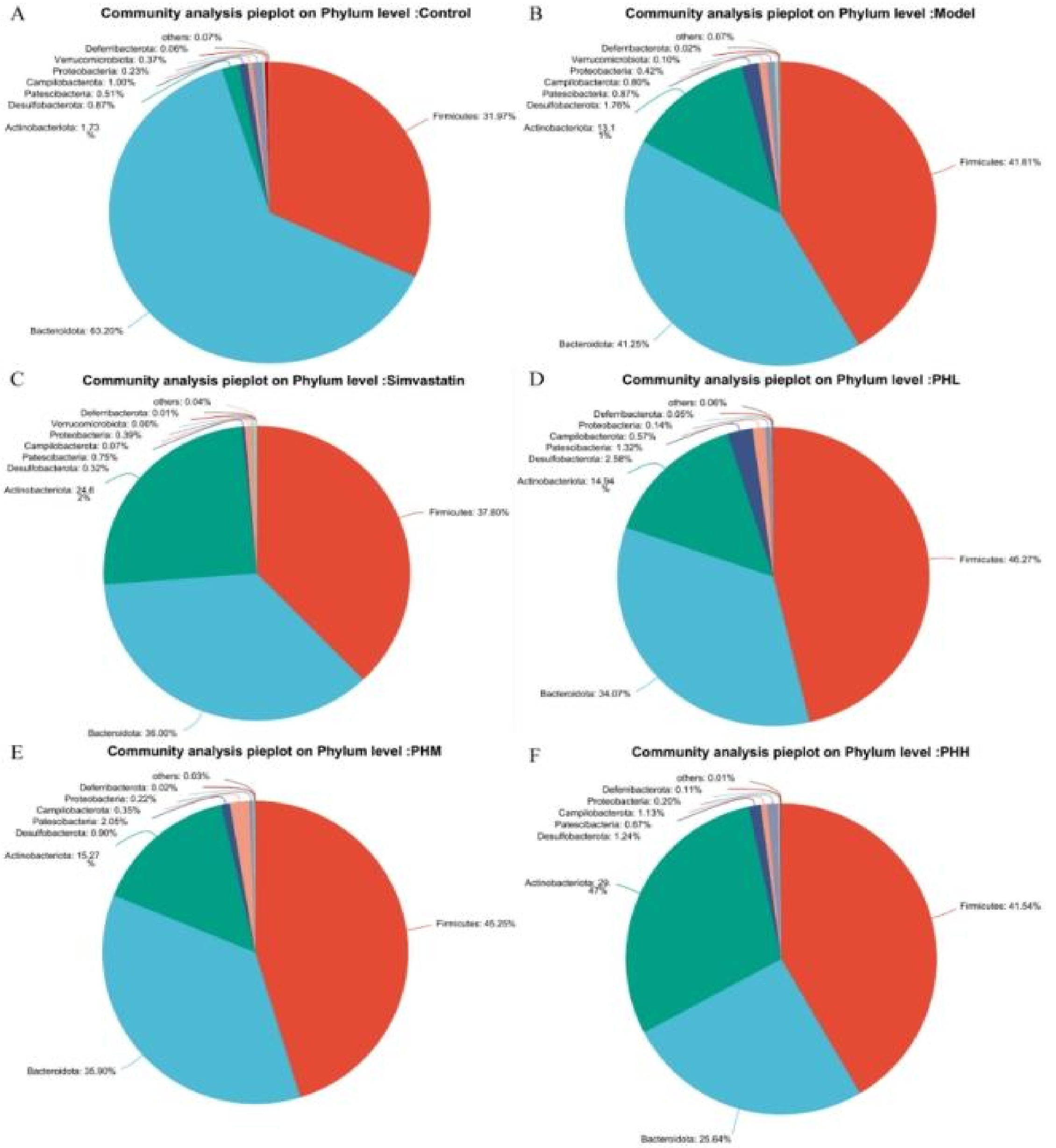

Figure S4. Venn analysis pie plot at phylum level

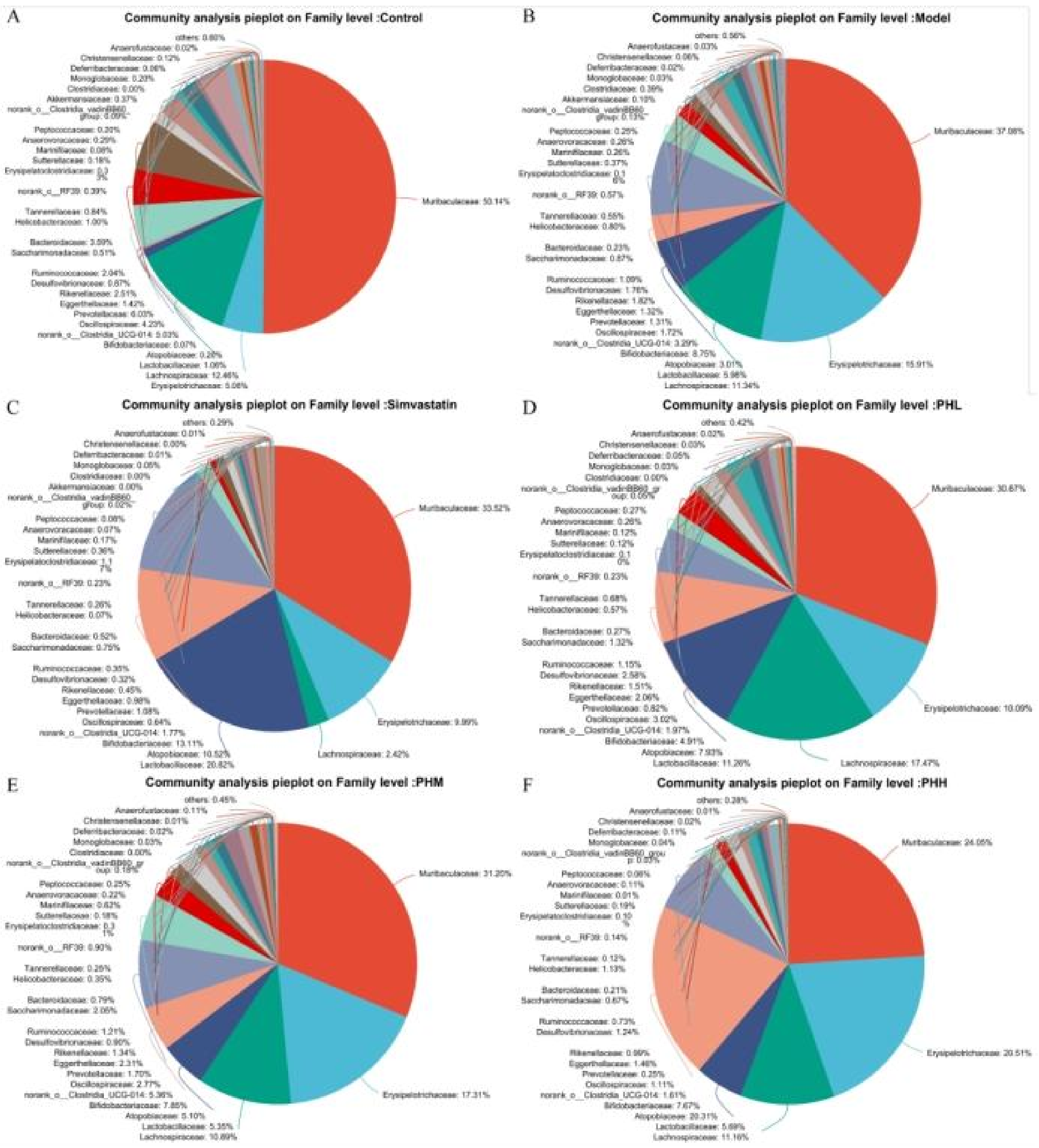

Figure S5. Venn analysis pie plot at family level

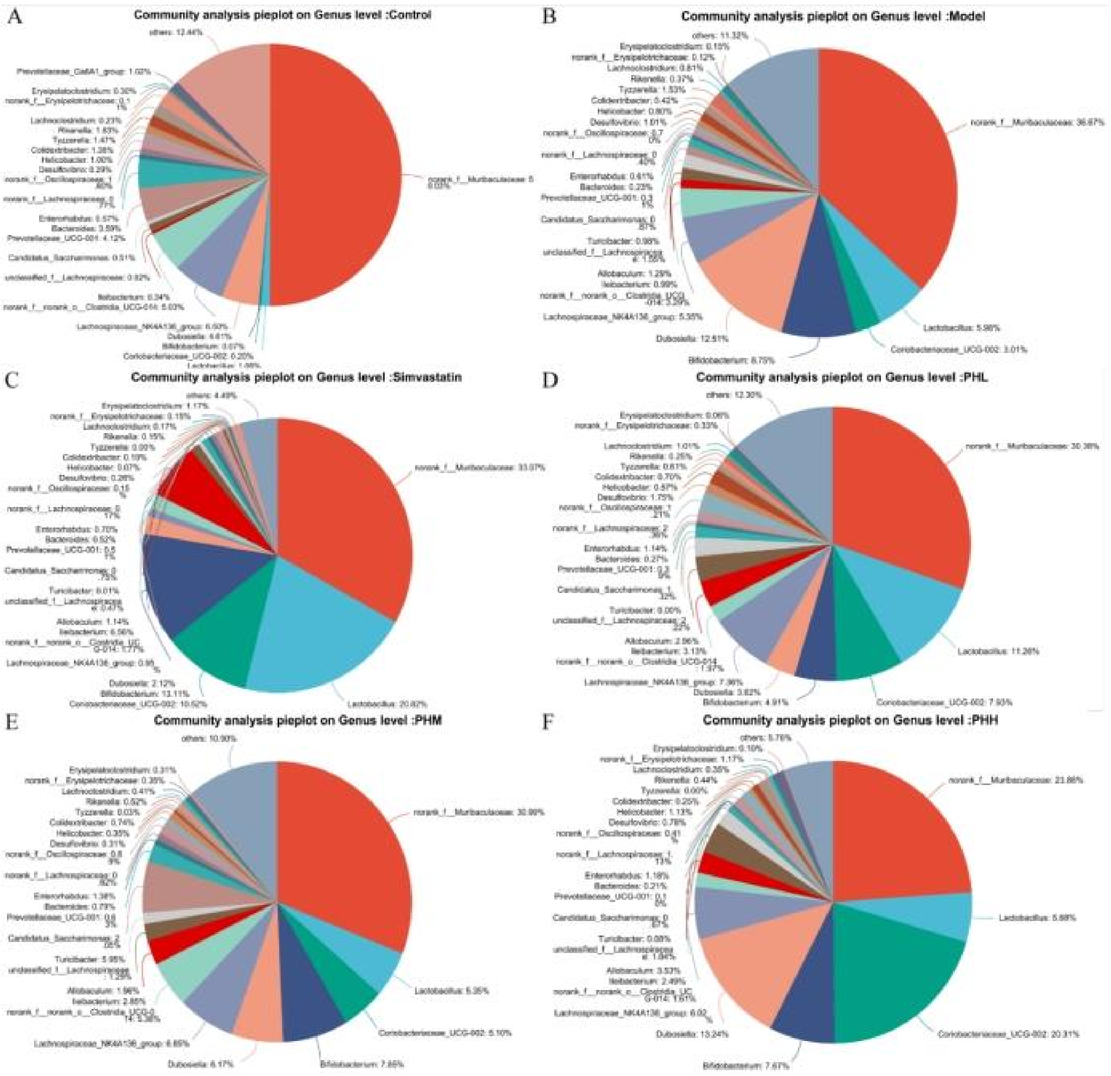

Figure S6. Venn analysis pie plot at genus level

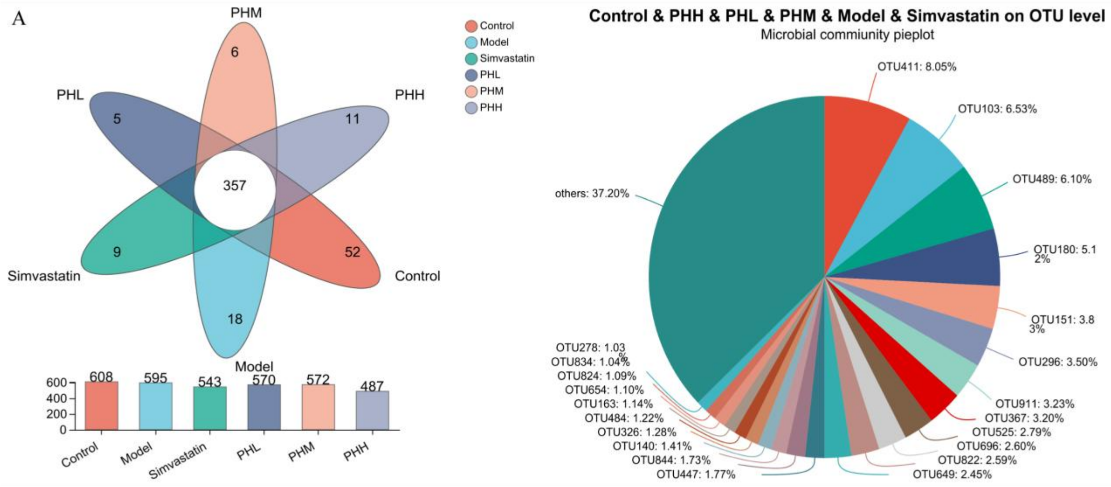

Figure S7. Microbial community at OTU level

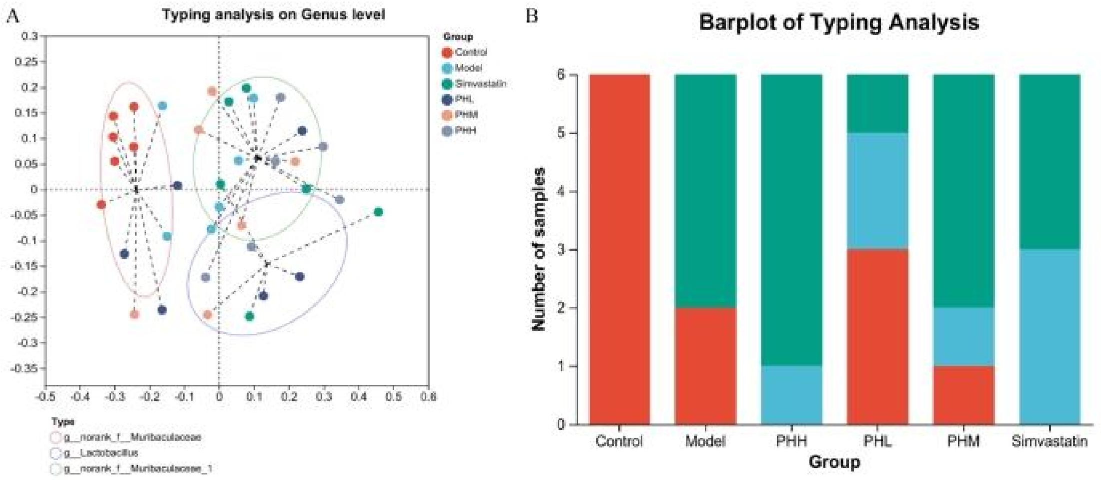

Figure S8. PCoA and barplot of typing analysis

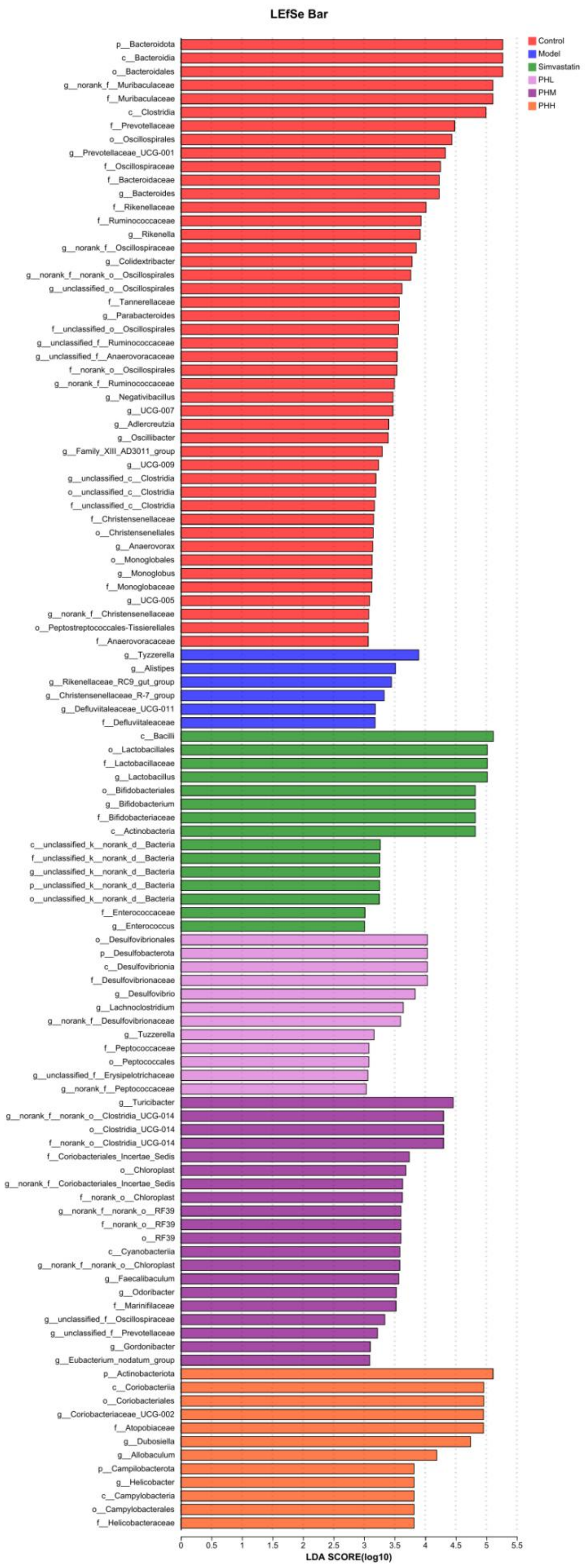

Figure S9. LEfSe analysis

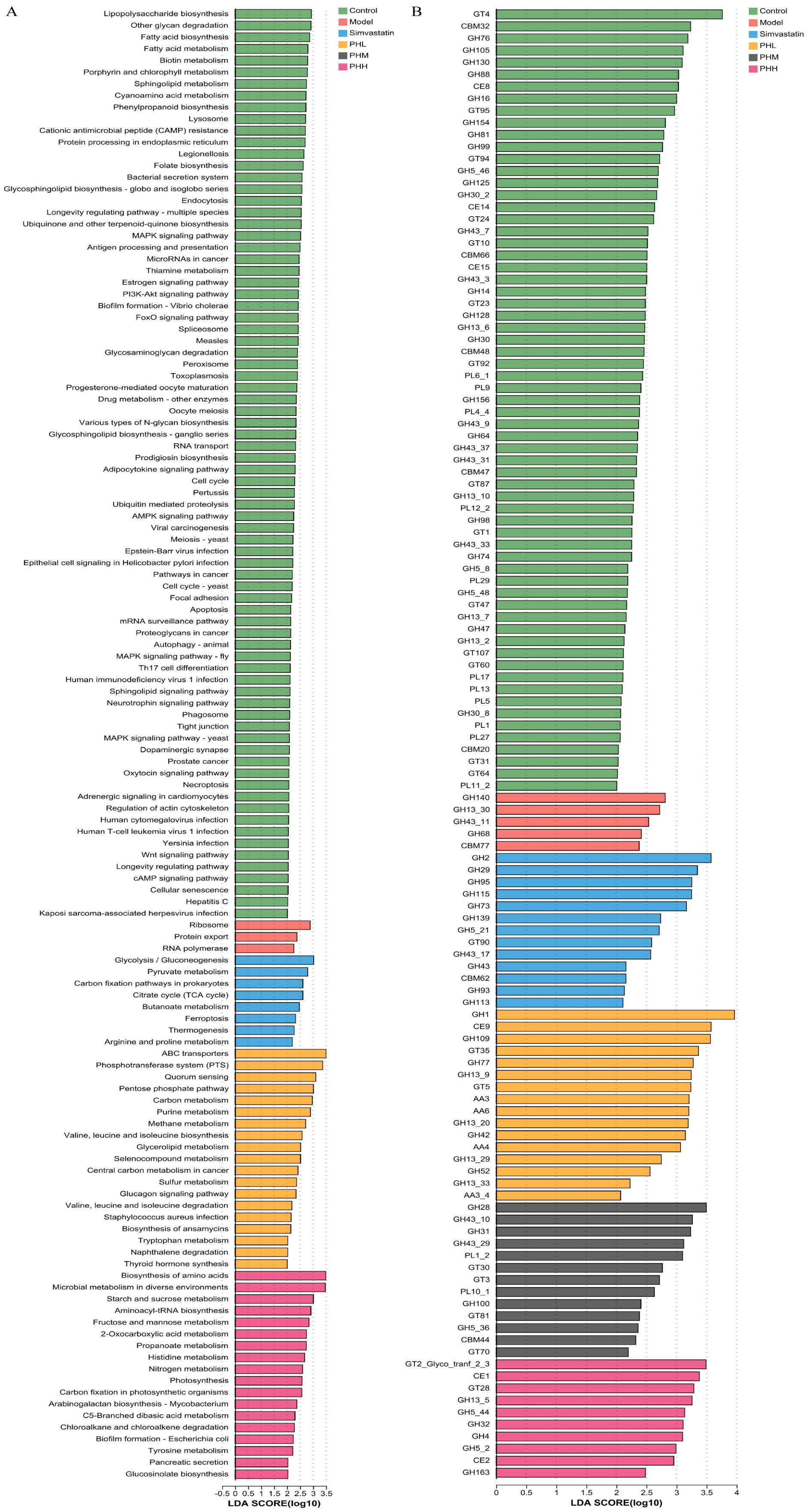

Figure S10. (A) LEfSe analysis of KEGG pathways (LDA>2.5); **(B)** LEfSe analysis of CAZymes

at the class level (LDA>3)

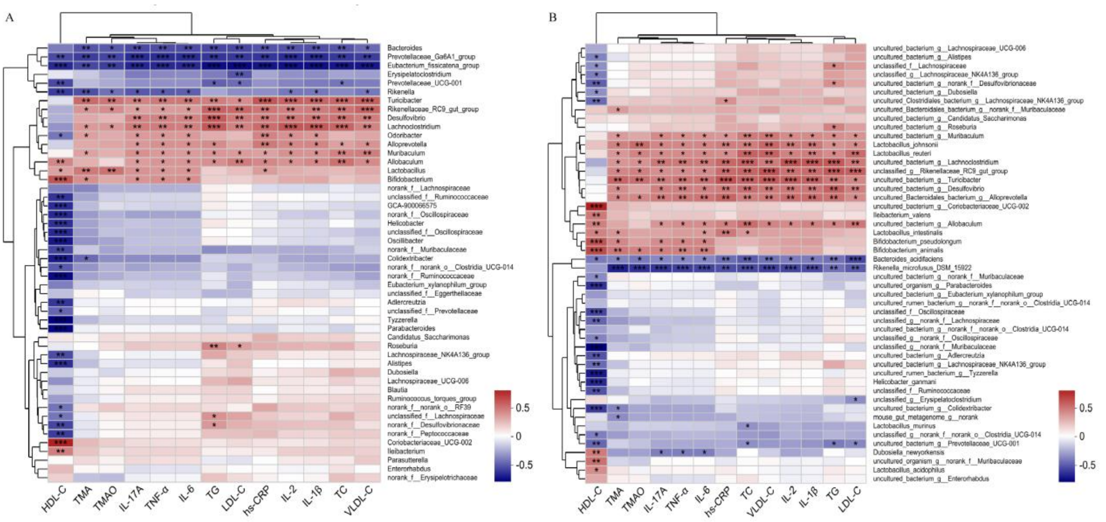

Figure S11. (A) and **(B)** Correlation heatmaps of gut microbiota and atherosclerotic parameters at the genus and species levels

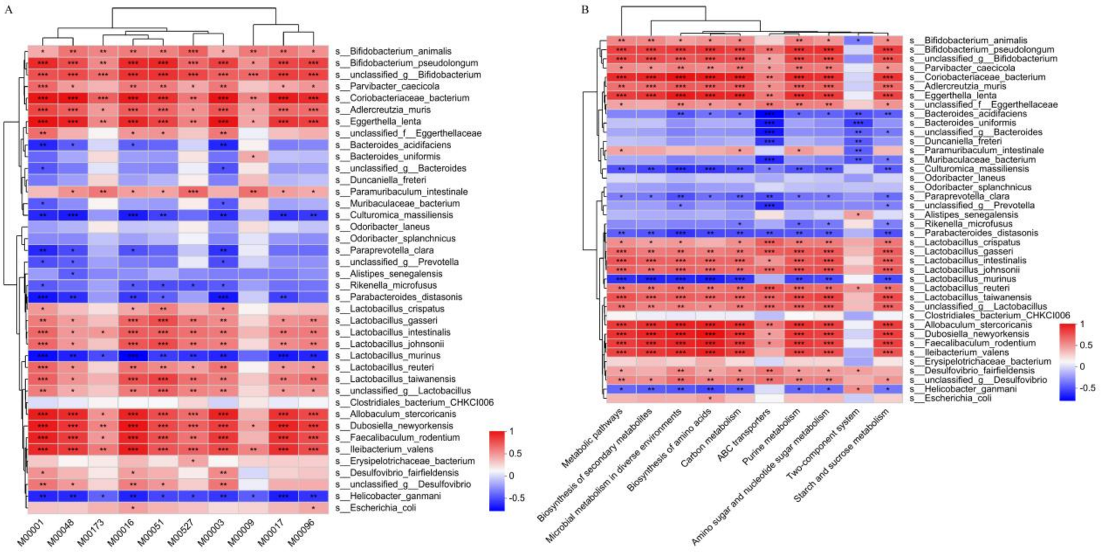

Figure S12. (A) and **(B)** Correlation between different species and KEGG functional pathways at modules and level 3. **P*<0.05, ***P*<0.01, ****P*<0.001.

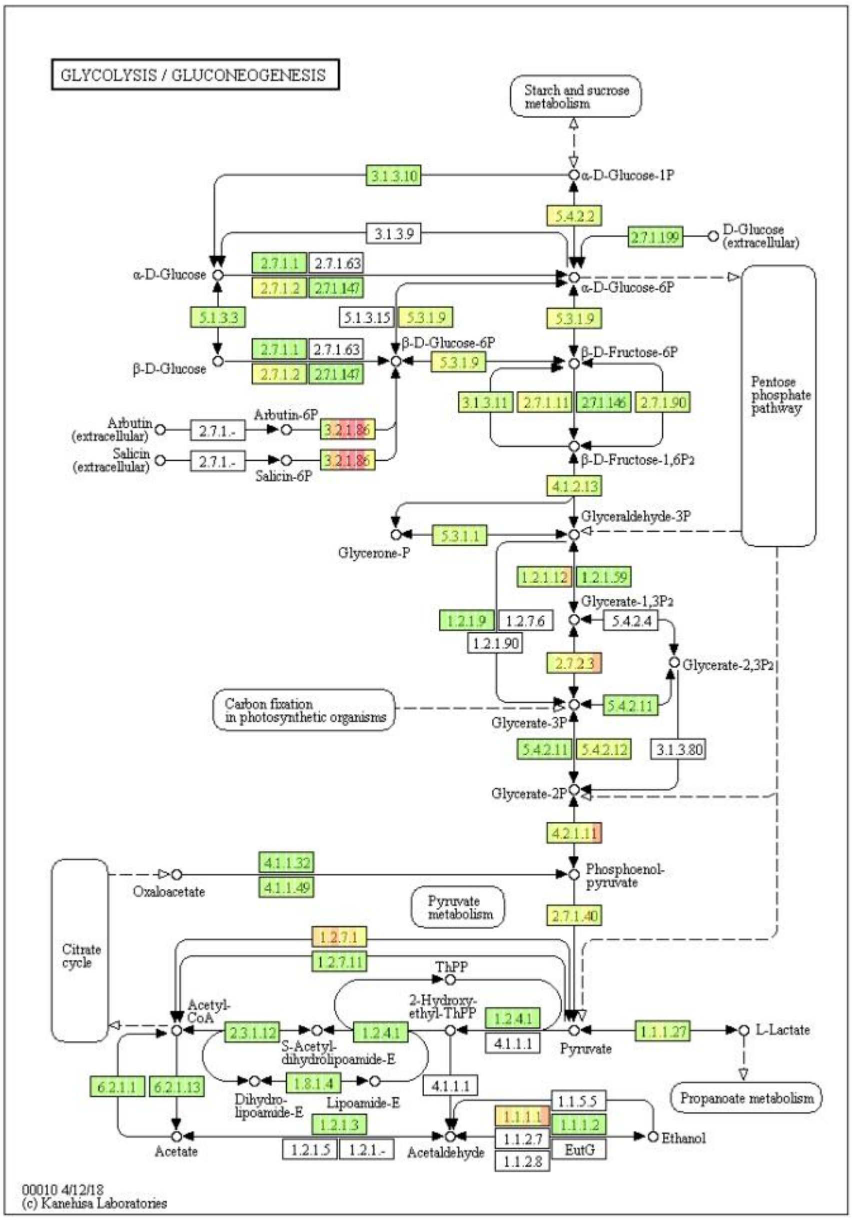

Figure S13. Glycolysis/Gluconeogenesis pathway

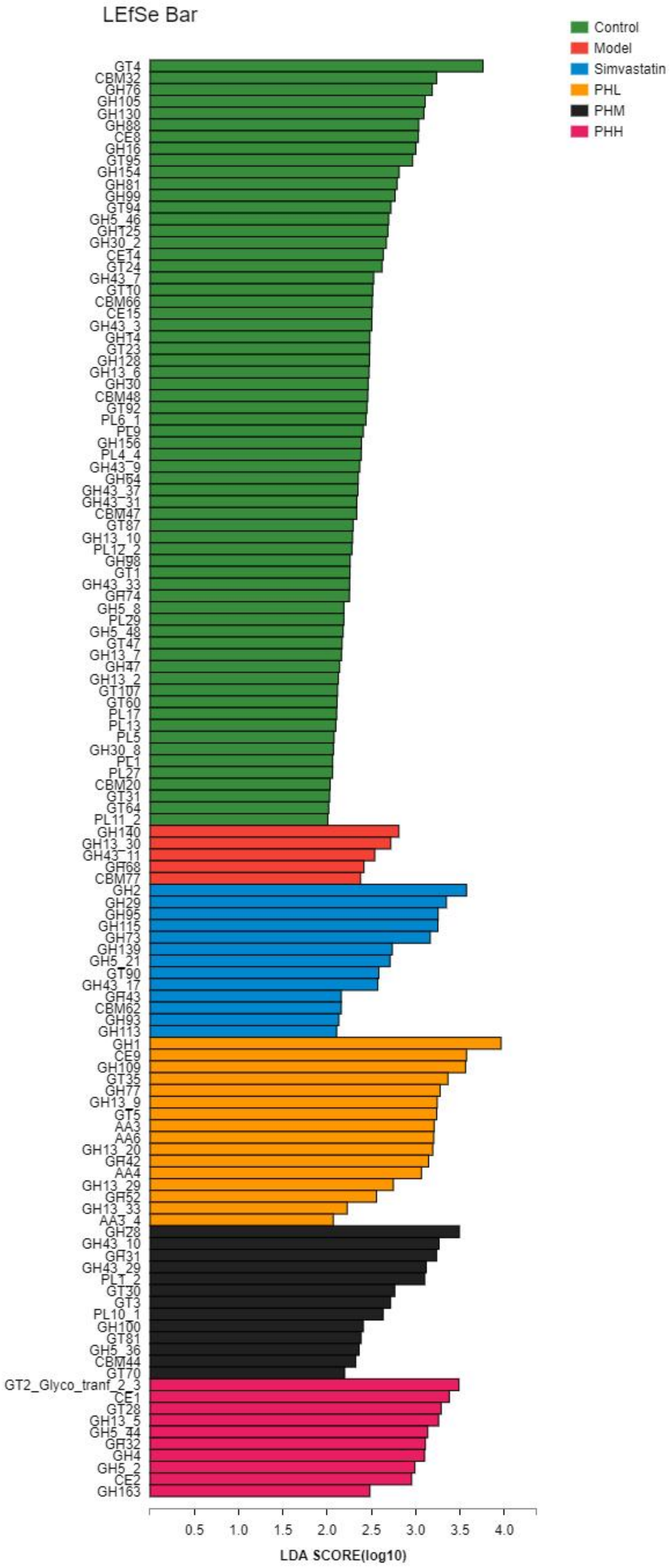

FigureS14. LEfSe analysis of the differential enrichment of CAZymes at the class level (LDA>3)
